# Characterization of human gastric carcinoma-related methylation of 9 *miR* CpG islands and repression of their expressions *in vitro* and *in vivo*

**DOI:** 10.1186/1471-2407-12-249

**Published:** 2012-06-15

**Authors:** Yantao Du, Zhaojun Liu, Liankun Gu, Jing Zhou, Bu-dong Zhu, Jiafu Ji, Dajun Deng

**Affiliations:** 1 Key Laboratory of Carcinogenesis and Translational Research (Ministry of Education), Division of Cancer Etiology, Peking University Cancer Hospital and Institute, Fu-Cheng-Lu, No.52, Haidian District, Beijing, 100142, China; 2 Department of Oncology, Peking University Cancer Hospital and Institute, Fu-Cheng-Lu, No.52, Haidian District, Beijing, 100142, China; 3 Department of Surgery, Peking University Cancer Hospital and Institute, Fu-Cheng-Lu, No.52, Haidian District, Beijing, 100142, China

## Abstract

**Background:**

Many *miR* genes are located within or around CpG islands. It is unclear whether methylation of these CpG islands represses *miR* transcription regularly. The aims of this study are to characterize gastric carcinoma (GC)-related methylation of *miR* CpG islands and its relationship with miRNA expression.

**Methods:**

Methylation status of 9 representative *miR* CpG islands in a panel of cell lines and human gastric samples (including 13 normal biopsies, 38 gastritis biopsies, 112 pairs of GCs and their surgical margin samples) was analyzed by bisulfite-DHPLC and sequencing. Mature miRNA levels were determined with quantitative RT-PCR. Relationships between *miR* methylation, transcription, GC development, and clinicopathological characteristics were statistically analyzed.

**Results:**

Methylation frequency of 5 *miR* CpG islands (*miR-9-1*, *miR-9-3*, *miR-137*, *miR-34b*, and *miR-210*) gradually increased while the proportion of methylated *miR-200b* gradually decreased during gastric carcinogenesis (*Ps* < 0.01). More *miR-9-1* methylation was detected in 62%-64% of the GC samples and 4% of the normal or gastritis samples (18/28 versus 2/48; Odds ratio, 41.4; *P* < 0.01). *miR-210* methylation showed high correlation with *H. pylori* infection. *miR-375*, *miR-203*, and *miR-193b* methylation might be host adaptation to the development of GCs. Methylation of these *miR* CpG islands was consistently shown to significantly decrease the corresponding miRNA levels presented in human cell lines. The inverse relationship was also observed for *miR-9-1*, *miR-9-3*, *miR-137*, and *miR-200b* in gastric samples. Among 112 GC patients, *miR-9-1* methylation was an independent favourable predictor of overall survival of GC patients in both univariate and multivariate analysis (*P* < 0.02).

**Conclusions:**

In conclusion, alteration of methylation status of 6 of 9 tested *miR* CpG islands was characterized in gastric carcinogenesis. *miR-210* methylation correlated with *H. pylori* infection. *miR-9-1* methylation may be a GC-specific event. Methylation of *miR* CpG islands may significantly down-regulate their transcription regularly.

## Background

miRNA are an abundant class of small non-coding RNAs that mainly regulate gene expression at the post-transcriptional level. They play critical roles in the renewal and differentiation of stem cells and help maintain cell lineages. Previous research has shown that in cancer several of the *miR* genes such as *miR-200b/200a/429*, *miR-21*, *miR-30b*, *miR-30d*, *miR-31*, and *miR-423* are upregulated, while other *miR* genes such as *miR-143* and *miR-145* are downregulated [1-3]. Evidence suggests that changes in miRNA expression occur frequently in many cancers and these variations either contribute to carcinogenesis or reflect the development and progression of cancers.

There are a number of pathways that may affect mature miRNA levels in cells and tissues, such as gene amplification or deletion, transcriptional upregulation or downregulation, post-transcriptional processing, and miRNA degradation [4-7]. It is well known that some intragenic *miR* genes, such as *miR-218-2*, are coordinately transcribed with their host genes through co-regulation mechanisms [8]. However, many *miR* genes are extragenic and a certain proportion of intragenic *miR* genes such as *miR-9-1* are transcribed in a host gene-independent pattern [9]. Because the exact promoter region of most *miR* genes are not characterized, especially with regard to the extragenic *miR* genes, the exact regulatory mechanisms of *miR* transcription are far from clear.

Methylation or hypermethylation of CpG islands in the region of transcription starting sites (TSS) is generally recognized to repress gene transcription epigenetically. Unlike protein-coding genes that may span multiple CpG islands, the *miR* genes may be shorter than a CpG island, and in some cases, multiple *miR* genes (i.e., a *miR* gene cluster) may be located within or flanking a single CpG island (Additional file 1: Table S1). Aberrant methylation of CpG islands associated with *miR* genes, such as *let-7a-3* and *miR-34a*, is frequently observed in many cancers [10,11]. It has been suggested that methylation of the CpG islands that are associated with *miR* genes (i.e. *miR-203*, *miR-152*, *miR-124-1*, *miR-34b/c*, *miR-129-2*, *miR-9-1*, *miR-130b*, *miR-124-2*, and *miR-181c*) might inversely correlate with their expression levels [12-17]. However, whether or not transcription of *miR* genes is regularly affected by the methylation status of *miR* CpG islands has not been systemically studied.

It is well known that abnormal methylation or demethylation of CpG islands in a small proportion (<1%) of the cell population can be sensitively detected in cellular heterozygous tissue samples. This demonstrates the advantage of methylation analysis over alterations of gene expression at the RNA and protein levels that can only be detected when such a changes is present in a large proportion of a cell population in a sample [18]. Our bioinformatic analysis shows 50, 9, and 70 of 721 human *miR* genes in the miRbase (Release 14.0) are located, respectively, within, flanking, and near CpG islands (collectively we will refer to these as *miR* CpG islands; Additional file 1: Table S1). We hypothesize that aberrant methylation of *miR* CpG islands may occur during development and progression of cancers. Therefore they could be used as candidate genes not only for prediction of cancer prognosis, but also for investigation of the methylation-expression association *in vivo*. Thus, CpG islands of 9 disease-related *miR* genes, including 5 extragenic *miR* genes or gene clusters (*miR-9-3*, *miR-137*, *miR-200b/200a/429*, *miR-203*, and *miR-375*) and 4 intragenic genes or gene clusters (*miR-9-1*, *miR-34b/c*, *miR-193b/365-1*, and *miR-210*), were selected as the representative genes in the present study (Additional file 1: Table S1). The methylation-expression association for 3 of these *miR* genes has not been previously established (Additional file 1: Table S2) [12,14,19-27]. We initially screened for gastric carcinoma (GC)- or host-related aberrant *miR* methylation and then investigated the methylation-expression association *in vitro* and *in vivo*. Associations between clinicopathological features of GC patients and methylation of these *miR* CpG islands were also analyzed.

## Methods

### Cell line sources and cell culture

Source information of used cell lines used in this study: RKO cell line, provided by Dr. Guoren Deng at University of California San Francisco; SW480 and HCT116 were provided by Dr. Yuanjia Chen at Peking Union Medical College Hospital; MKN74 and 293 T provided by Tokyo Medical and Dental University; PC-3 was purchased from the Cell Line Bank at the Chinese Academy of Medical Science; HL60 and KG1A were obtained from the Hematology Department of Peking University First Hospital; Du145 was obtained from Hanmi Pharmacy Company; Siha was provided by Peking University People’s Hospital. HepG2 was provided by Dr. Qingyun Zhang, Calu3 and A549 by Dr. Zhiqian Zhang, H1299 and AGS by Dr. Chengchao Shou, MKN45 by Dr Youyong Lv, and other cell lines (SGC7901, BGC823, MGC803, HeLa and GES-1) by Dr. Yang Ke, all at Peking University Cancer Hospital/Institute. These cell lines were cultured at 37°C in 5% CO_2_, using various culture media. MKN45, MKN74, SGC7901, BGC823, MGC803, HL60, KG1A, A549, H1299, GES-1, HepG2, 293 T, Du145, and RKO were cultured in 90% RPMI-1640 and 10% FBS. PC-3 and AGS were cultured in 90% F-12 and 10% FBS. Calu3, HeLa and Siha were cultured in 90% DMEM and 10% FBS. SW480 and HCT116 were cultured in 90% DMEM:RPMI-1640 (1:1) and 10% FBS.

### Patients and tissue samples

Surgical primary GC samples and their paired non-cancerous surgical margin (SM) samples were collected from 112 inpatients (average age 59.2 years [range, 32–79]; 80 males and 32 females; 78 non-cardiac GCs and 34 cardiac GCs; 40 GCs at pTNM stage I ~ II and 59 at the stage III ~ IV) at Peking University Cancer Hospital. Follow-up data for all patients was collected for at least five years. All clinical samples, as well as histopathological and followup information for each case were obtained according to approved institutional guidelines. Gastric biopsies from 13 healthy subjects and 38 gastritis outpatients collected from the same hospital were used as non-cancer patient controls. Before bisulfite modification each patient’s gastric genomic DNA sample was analyzed for the presence of *H. pylori*-specific *23 S rDNA* by a PCR assay as described previously [28]. The Institutional Review Boards of Peking University Cancer Hospital and Institute approved the study (#2011041207), and all patients gave written informed consent.

### DNA extraction and bisulfite modification

Cancer cell line and tissue sample genomic DNA (1.8 μg) was isolated using phenol/chloroform extraction [29]. The unmethylated cytosine residues in the DNA samples were converted to uracil residues (becoming thymidine residues in PCR products) by the addition of 5 M sodium bisulfite [30]. The Wizard® DNA Clean-Up System Kit (Promega) was used to purify the bisulfite-treated DNA before PCR amplification.

### PCR amplification and quantification of *miR* CpG island methylation by DHPLC

CpG-free universal primer sets were used to amplify *miR* CpG islands by hot-start PCR polymerase (Additional file 1: Figure S1 and Additional file 1: Table S3). Sequences of CpG islands embedded or flanked these related *miR* genes were used to design the primers. The PCR products were then analyzed quantitatively by DHPLC on the WAVE® DNA Fragment Analysis System [31]. Elution profiles of *miR-9-3*, *miR-200b*, and *miR-203* methylation was analyzed with an ultraviolet detector; other *miR* gene methylation was detected with the post column HSX-3500 Accessory (Transgenomic, Inc., Omaha, USA) and a high–sensitivity fluorescence (FL) detector (excitation at 450 nm, emission at 520 nm) [32]. Methylated and unmethylated *miR* gene PCR products were separated by a DNASep® analytical column (Transgenomic) at the corresponding partial denaturing temperature (Additional file 1: Table S3 and Figure S2-10). The peak areas corresponding to the methylated and unmethylated PCR products were used to calculate the proportion of methylated *miR* CpG island [the proportion of methylated copies = methylation-peak area/ total peak area] as previously described [33]. *M.SssI-*methylated genomic DNA from blood samples was used as a positive control.

**Figure 1:**
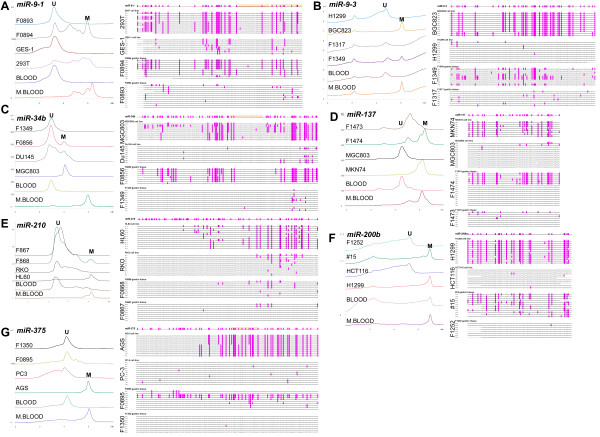
**DHPLC chromatograms and bisulfite sequencing of 7 *miR *CpG islands in representative cell lines and gastric tissue samples.** Both human peripheral blood DNA (BLOOD) and its M.*Sss*I-methylated products (M.BLOOD) were used as controls. All CpG sites in the amplicon of each *miR* CpG island are also listed above the results of bisulfite sequencing. Each row represents one clone; each pink bar represents a methylated CpG site. The corresponding chromatogram of each sequenced sample (right column) is shown (left column).

### Bisulfite clone-sequencing

Fresh PCR products of *miR* CpG islands amplified with the CpG-free universal primer sets were cloned with the pGEM-T Easy kit (Promega, Madison, USA) and sequenced with an Applied Biosystems 3730xl DNA Analyzer at SinoGeneMox Company (Beijing, China).

### Extraction of RNA and detection of mature miRNA level with quantitative RT-PCR assays

Total 50 ng RNA was extracted from fresh tissue samples or cell lines using the TRIzol reagent (Life Technologies, Carlsbad, USA) according to the manufacturer’s protocol. Corresponding cDNA samples were synthesized using the TaqMan® MicroRNA Reverse Transcription Kit (Life Technologies) with *miR*-specific stem-loop inverse transcription (RT) primers (specific for *miR-375* #RT000564, *miR-34b* #RT000427, *miR-137* #RT000593, and *miR-9* #RT000583). The RT conditions used were 16°C for 30 min ➔ 42°C for 30 min ➔ 85°C for 5 min. The miRNA levels were then analyzed using a TaqMan Gene Expression Master Mix kit (Life Technologies) with the corresponding probe and primers (Life Technologies, *miR-375* #TM000564, *miR-34b* #TM000427, *miR-137* #TM000593, and *miR-9* #TM000583). *U6* (Life Technologies, #RT001093 and #TM001093) was used as the internal reference. The PCR cycling conditions were 95°C for 10min ➔ followed by 40 cycles of 95°C for 20 sec ➔ 60°C for 1 min. Expression levels of *miR-200b* and *miR-210* were determined using a standard polyA RT-PCR assay. Sequences of the RT adaptor primer, the universal inverse primer and the *U6* primer in the regular polyA RT-PCR assay are shown in Additional file 1: Table S5. RT conditions were 55°C for 5 min ➔ followed by 25°C for 10 min ➔ 42°C for 1 hr ➔ and 70°C for 5 min. The PCR cycling conditions were 95°C for10min ➔ followed by 40 cycles of 95°C for 20 sec ➔ and 61°C for 1 min.

### Statistical analysis

SPSS 16.0 *Trend*-test and Pearson’s Chi-square test were used to analyze the *miR* methylation frequency difference between normal biopsies, gastritis lesions, GC and SM samples. Kruskal-Wallis *H*-test and One-Way ANOVA were used to analyze the *miR* methylation proportion differences between normal biopsies, gastritis lesions, GC, and SM samples. Fisher’s exact test, Pearson’s Chi-square test, and *Trend*-test were used to analyze the association between *miR* methylation positive rates and the clinicopathological features. The Mann–Whitney *U*-test and Student’s *t*-test were used to analyze the association between the proportion of methylated *miR* alleles and the clinicopathological features. Kaplan-Meier and Cox-Proportional Hazards methods were used for univariate and multivariate analysis to compare overall survival of GC patients with differences in methylation status of *miR* CpG islands. All statistical tests were two-sided, and *P* < 0.05 was considered statistically significant.

## Results

### Characterization of methylation or demethylation of 6 *miR* CpG islands related to the development of GCs

We amplified bisulfite-treated templates of 9 representative *miR* CpG islands with CpG-free primers and developed 9 DHPLC assays to analyze the methylation status of the CpG islands in PCR products of 4 intragenic *miR* genes (*miR-9-1*, *miR-34b*, *miR-193b*, *miR-210*) and 5 extragenic *miR* genes (*miR-9-3*, *miR-137*, *miR-200b*, *miR-203* and *miR-375*), respectively (Figure 1, Additional file 1: Table S3, and Additional file 1: Figure S1–S10). These DHPLC assays revealed that the methylation positive rate of 5 *miR* CpG islands (*miR-9-1*, *miR-9-3*, *miR-34b*, *miR-137*, and *miR-210*) was significantly increased concurrently with the severity of pathological changes in the stomach. These findings strongly suggest that methylation of these *miR* CpG islands is related to the development of GCs (*trend*-test, *miR-9-1*, *P <* 0.001; *miR-9-3*, *P <* 0.001; *miR-34b*, *P =* 0.008; *miR-137*, *P <* 0.001; *miR-210*, *P* = 0.001; Table 1). *miR-9-1* methylation was detected in 18 of 28 GCs (sensitivity, 64%), but only in 2 of 48 normal or gastritis biopsies showed methylation (specificity, 96%). Although methylation of *miR-200b* was detected in almost all gastric tissues samples, the proportion of methylated *miR-200b* in normal or gastritis tissues (46% ~ 100%) was significantly higher than that in both SM and GC samples (41% ~ 47%) (Mann–Whitney *U-*test, gastritis biopsies versus SMs, *P =* 0.001; Table 1). This suggests that *miR-200b* demethylation was a GC, patient-specific event. Bisulfite sequencing of these *miR* CpG islands confirmed the data obtained from DHPLC analysis (Figure 1).

**Table 1:** **Methylation status of *miR *CpG islands in gastric mucosa samples with different pathological changes in GC and non-cancerous control patients**

***miR *CpG islands**	**Group of gastric samples**	***miR *-Methylation**						
		**Positive rate**			**Proportion (%) of methylated *miR *in the *miR *methylation-positive samples**			
		**Positive rate (%)**	***χ***^***2***^**-value**	***Trend*-test** (***P*-value)**	***Median*[25% ~ 75%]**	***Mean*±*SD***	***t/F/χ ***^***2***^**-value**	***P*-value**
*miR-9-1*	Normal	1/13 (7.7)	27.598	<0.001 ^**a**^	NA			NA
	Gastritis	1/35 (2.9)			NA			
	SM	9/28 (32.1)			17 [13–30]	20 ± 4	*t* = −3.039	0.005 ^**g**^
	GC	18/28 (64.3)			29 [19–40]	32 ± 4		
*miR-9-3*	Normal	6/13 (46.2)	23.389	<0.001 ^**a**^	38 [31–59]	43 ± 6	*F* = 1.639	0.189 ^**h**^
	Gastritis	15/37 (40.5)			43 [36–48]	42 ± 2		
	SM	26/28 (92.9)			38 [26–44]	36 ± 2		
	GC	26/28 (92.9)			37 [26–43]	36 ± 2		
*miR-137*	Normal	5/13 (38.5)	18.626	<0.001 ^**a**^	10 [4–41]		*χ*^*2*^ = 8.065	0.045 ^**d**^
	Gastritis	24/38 (63.2)			19 [7–36]			
	SM	25/26 (96.4)			15 [7–40]	21 ± 3^g^		
	GC	24/26 (92.3)			36 [20–51]	37 ± 4		
*miR-34b*	Normal	6/13 (46.2)	6.947	0.008 ^**a**^	13 [7–19]		*χ*^*2*^ = 0.658	0.883 ^**d**^
	Gastritis	13/36 (36.1)			11 [3–20]			
	SM	22/28 (78.6)			11 [4–18]			
	GC	19/28 (67.9)			10 [5–32]			
*miR-200b*	Normal	13/13 (100)	0.486	0.922 ^**f**^	54 [52–83]		*χ*^*2*^ = 17.883	<0.001 ^**d**^
	Gastritis	35/36 (97.2)			52 [46–100]^**e**^			
	SM	27/28 (96.4)			44 [40–47]			
	GC	27/28 (96.4)			48 [40–53]			
*miR-210*	Normal	2/13 (15.4)	11.908	0.001 ^**a**^	NA			NA
	Gastritis	13/38 (34.2)			7 [5–10]			
	SM	22/27 (81.5)			11 [5–22]			
	GC	16/27 (59.3)			9 [4–16]			
*miR-193b*	Normal	0/13	7.045	0.008 ^**b**^	NA			NA
	Gastritis	2/37 (5.4)			NA			
	SM	7/28 (25.0)			5 [4–14]			
	GC	4/28 (14.3)			5 [2–12]			
*miR-203*	Normal	5/13 (38.5)	5.617	0.018 ^**b**^	32 [29–75]		*χ*^*2*^ = 2.957	0.398 ^**d**^
	Gastritis	20/38 (52.6)			31 [26–36]			
	SM	21/28 (75.0)			34 [30–36]			
	GC	11/28 (39.3)^c^			32 [29–35]			
*miR-375*	Normal	0/13	12.266	<0.001 ^**b**^	NA			NA
	Gastritis	13/38 (34.2)			3 [3–4]^**e**^			
	SM	16/28 (57.1)			7 [4–14]			
	GC	8/28 (28.6) ^**c**^			17 [8–21]			

^a,^*Trend* test, among Normal, Gastritis, SM and GC samples; ^b,^*Trend*-test, among Normal, Gastritis, and SM samples; ^c,^ Pearson’s Chi-square test, GC versus SM: *miR-203*, *χ*^*2*^ = 7.292, *P =* 0.007; *miR-375*, *χ*^*2*^ = 4.667, *P =* 0.031; ^d,^ Kruskal-Wallis *H*-test; ^e,^ Mann–Whitney *U*-test: Gastritis versus SM, *miR-200b*,*U* = 230.000, *P =* 0.001; Gastritis versus GC, *miR-375*, *U* = 46.000, *P =* 0.011; SM versus GC, *miR-137*, *U* = 170.000, *P* = 0.009; ^f,^ Pearson’s Chi-square test, among Normal, Gastritis, SM and GC samples; ^g.^ Paired *t*-test: SM versus GC, *miR-137*, *t* = −2.652, *P =* 0.014; ^h.^ One-way ANOVA; NA, not available.

Except for *miR-9-1* and *miR-137*, the methylation positive rates or proportions of methylated *miR-9-3*, *miR-34b*, *miR-210*, and *miR-200b* in GCs were similar to those in SMs (Table 1). To validate if methylation of some of these *miR* genes is a field-effect happens simultaneously in both cancerous and non-cancerous tissues in the stomach due to the same exposure to environmental factors, we detected the methylation data in another subset of GC and SM samples from 84 patients and found that the positive rate and proportion of methylated *miR-9-1* in GCs was still significantly higher than that in SMs (60.7% versus 32.1%; Pearson’s Chi-square test, *P =* 0.001; Sign rank test, *P* < 0.001; Additional file 1: Table S4, Subset-2). The average proportion of the methylated *miR-137* was also significantly higher in GCs than SMs (*Mean* ± *SD*, 26 ± 2 versus 38 ± 2, Paired *t-*test, *P <* 0.001). As expected a significant difference in the positive rate or the proportion of methylated *miR-9-3, miR-34b*, *miR-210*, and *miR-200b* was not observed between GC and SM samples. These results confirmed that *miR-9-1* and *miR-137* methylation was a tumor-specific event and that *miR-9-3*, *miR-34b*, and *miR-210* methylation, as well as *miR-200b* demethylation, was a field-effect that occurred during gastric carcinogenesis.

Furthermore, the positive rate of methylated *miR-203* and *miR-375* gradually increased from normal to gastritis to SM samples, but significantly decreased in GCs compared with SMs (Pearson Chi-square test, *miR-203*, *P =* 0.007; *miR-375*, *P =* 0.031; Table 1). In the Subset-2 samples, we also analyzed *miR-375* methylation and found more *miR-375* methylation in SMs than in GCs again (Pearson Chi-square test, *P =* 0.034; Additional file 1: Table S4). These data imply that *miR-375* (and *miR-203*) methylation is not GC-specific and might be one kind of host adaptation in the non-malignant tissues to the development of GCs.

### *miR* methylation and *H. Pylori* infection

**Figure 2:**
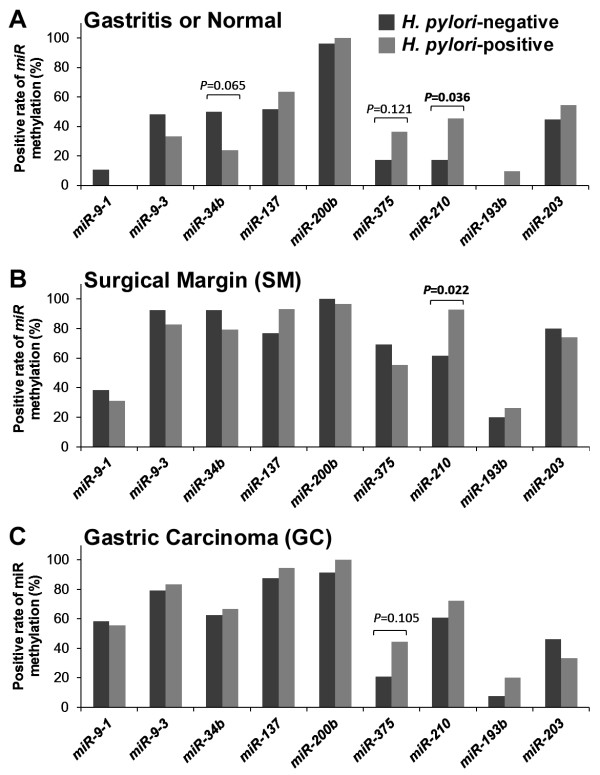
**Comparison of *miR *methylation in various gastric mucosa samples with and without existence of *H. pylori.*** (**A**) Normal or gastritis biopsies from control outpatients without malignant disease; (**B** and **C**) Surgical margin and tumor samples from patients with gastric carcinoma, respectively; *H. pylori-specific 23 S rDNA* was detected by PCR.

To determine if *H. pylori* infection plays a role in *miR* methylation, we looked for *H. pylori*-specific *23 S rDNA* in gastric genomic DNA samples by PCR and found that the *H. pylori*-positive rate increased concurrently with the severity of pathological changes [15.4% (2 of 13) of normal gastric biopsies, 52.6% (20 of 38) of gastritis lesions, and 69.0% (29 of 42) SM biopsies; *trend*-test, *P =* 0.001] and decreased in GC samples (18 of 42 = 42.9%; GCs versus SMs, Pearson Chi-square test, *P* = 0.016; Additional file 1: Figure S11). *H. pylori*-positive gastritis/normal and SM samples showed significantly higher methylation of *miR-210* methylation than *H. pylori*-negative samples (*P* = 0.036 and 0.022, respectively; Figure 2A and B).

### Inversed relationship between *miR* methylation of CpG islands and their corresponding expression levels

**Figure 3:**
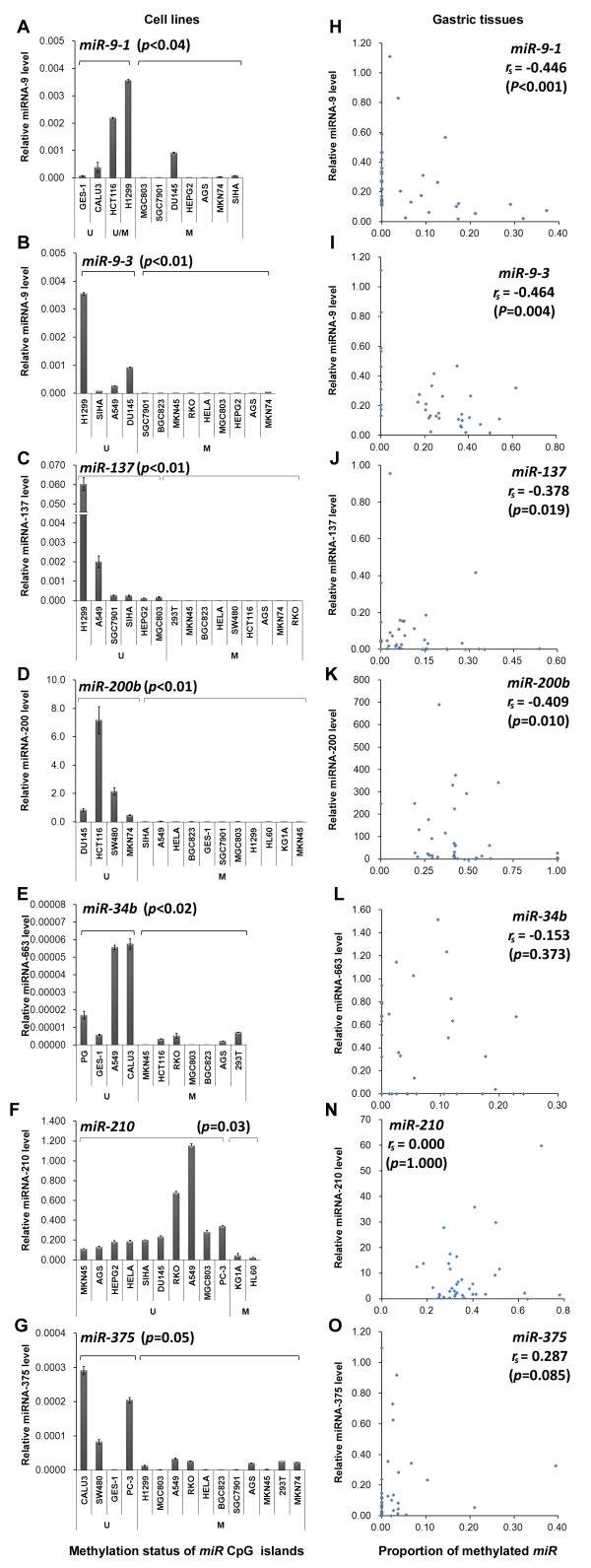
**The relationship between expression level and methylation status of *miR *genes in human cell lines and gastric tissue samples.** (**A-G**) The correlation analysis between methylation proportion of miRNA CpG islands and their mature miRNA expression levels in the target CpG island methylated and unmethylated human cell lines; (**H-P**) Expression of miRNAs in 20 paired, fresh gastric carcinoma samples; (**U**) Target CpG island unmethylated cell lines; (**M**) Target CpG island methylated cell lines; (**U/M**) Target CpG island partially methylated cell lines.

To investigate the relationship between the above aberrant *miR* methylation and the transcription of the corresponding *miR* gene, we quantified the mature miRNA levels of *miR-9-1*, *miR-9-3*, *miR-34b*, *miR-137*, *miR-210*, *miR-200b*, (and *miR-375*), whose methylation status is related to the development of GC (and GC host adaptation) as described above, in a set of human cell lines with different methylation status of *miR* CpG islands. The methylation status of each *miR* CpG island in these cell lines was determined by DHPLC as illustrated in Additional file 1: Figure S2–S10. Results of quantitative RT-PCR assays showed that in the tested cell lines containing methylated *miR* alleles the mature miRNA levels of all 6 GC-related *miR* genes and one host adaptation *miR* gene were significantly lower than those detected in the cell lines containing unmethylated *miR* alleles (Figure 3A-G).

We further analyzed the miRNA levels of 20 pairs of fresh GC and SM samples and found a significantly higher expression level of miRNA-200b*,* miRNA-375, and miRNA-210 in SMs than in GCs (Paired *t*-test: *Ps*≤0.030; Additional file 1: Figure S12). The miRNA-137 levels in SMs were also higher than those in GCs, but was not statistically significant (*P =* 0.059). The expression levels of miRNA-9 and miRNA-34b were similar between SMs and GCs. Most importantly, an inverse relationship between *miR* methylation and the corresponding expression level was observed for *miR-9-1*, *miR-9-3*, *miR-137*, and *miR-200b* in these gastric tissue samples (Spearman’s Rank Correlation analysis, *miR-9-1*, *r*_*s*_ = −0.533, *P =* 0.001; *miR-9-3*, *r*_*s*_ = −0.464, *P =* 0.004; *miR-137*, *r*_*s*_ = −0.378, *P =* 0.019; *miR-200b*, *r*_*s*_ = −0.409, *P =* 0.010; Figure 3H-K). A weak inverse methylation-expression relationship was also found for *miR-375* in these tissue samples (*r*_*s*_ =0.287, *P =* 0.085; Figure 3O). Such a relationship was not observed for *miR-34b* and *miR-210* (Figure 3L, N).

### *miR* methylation correlated to clinicopathological characteristics of GC patients

To study the possibility of using *miR* methylation as a prognosis predictor of GCs, we determined the prevalence of methylation of 7 *miR* genes and analyzed the relationship between the clinicopathological features of GC and the corresponding methylation levels of these *miR* CpG islands studied above for all 112 GC patients (Table 2 and Additional file 1: Table S6). Higher *miR-9-1* methylation was consistently observed in well/moderately differentiated, non-metastatic/less-invasive GCs, but this was not statistically significant. The proportion of demethylated *miR-200b* was higher in lymph metastatic GCs than in non-metastatic GCs (*median*, 45% versus 53%; Mann-Whiney *U*-test, *P =* 0.021). In contrast, the positive rate of *miR-137* methylation positively correlated with the depth of invasion, pTNM, lymph metastasis, and vessel embolus of GCs (*trend*-test for depth of invasion, *P =* 0.012; Pearson’s Chi-square test for pTNM/ lymph metastasis/ vascular embolus, *P =* 0.024/ 0.028/ 0.031). Similarly, higher *miR-210* methylation was observed in GCs with distant metastasis than in GCs without distant metastasis (*P =* 0.048). In addition, the positive rate of *miR-9-1* methylation and the proportion of methylated *miR-137* in elderly GC patients were significantly higher than in younger patients (Pearson’s Chi-square test, *miR-9-1*, *P =* 0.005; Student’s *t*-test, *miR-137*, *P =* 0.009).

**Table 2:** **Comparison of methylation of 7 *miR *CpG islands in 112 GC samples from patients with various clinicopathological characteristics**

**Clinicopathological features**		***miR-9-1 *methylation**		***miR-9-3 *methylation**		***miR-137 *methylation**		***miR-34b *methylation**		***miR-200b *methylation**		***miR-375 *methylation**		***miR-210 *methylation**	
		**Positive rate (%)**	Proportion, Median * [25-75%]	**Positive rate (%)**	**Proportion, *Median *[25-75%]**	**Positive rate (%)**	**Proportion, *Median *[25-75%]**	**Positive rate (%)**	**Proportion, *Median *[25-75%]**	**Positive rate (%)**	**Proportion, *Median *[25-75%]**	**Positive rate (%)**	**Proportion, *Median *[25-75%]**	**Positive rate (%)**	**Proportion, *Median *[25-75%]**
Age	≤60 (*n* = 49)	46.9^**a**^	21 [14–38]	68.8	35 [31–42]	95.9	34 [16–48]^**b**^	62.5	5 [2–16]	92.9	49 [43–55]	54.3	3 [2–14]	65.1	9 [5–27]
	>60 (*n* = 63)	73.0	33 [18–44]	61.0	36 [30–44]	93.3	47 [29–61]	54.2	10 [3–23]	87.7	48 [40–56]	38.3	3 [2–14]	72.9	10 [5–17]
Sex	Male (*n* = 80)	61.2	31 [14–45]	62.7	35 [30–42]	96.1	40 [24–54]	53.8	7 [3–18]	88.4	48 [40–56]	44.6	3 [2–7]	69.9	9 [5–17]
	Female (*n* = 32)	62.5	27 [17–36]	68.8	41 [30–43]	90.6	43 [18–54]	69.0	8 [2–26]	93.3	51 [46–55]	46.9	8 [2–19]	69.0	12 [5–22]
Preoperative chemotherapy	No (*n* = 100)	63.0	32 [16–44]	63.2	35 [30–43]	93.8	39 [23–56]	58.9	7 [3–18]	91.0	49 [41–56]	44.7	4 [2–16]	69.6	9 [5–19]
	Yes (*n* = 9)	55.6	21 [12–31]	66.7	37 [31–54]	100	49 [30–53]	44.4	5 [2–16]	71.4	49 [42–76]	44.4	2 [2–4]	57.1	13 [10–24]
Location	Cardiac (*n* = 34)	64.7	37 [21–47]	65.6	43 [35–51]^**b**^	90.9	49 [28–61]^**b**^	57.6	8 [3–23]	75.0^**d**^	43 [38–52]	31.2	3 [1–8]	64.5	9 [4–17]
	Non-cardiac (*n* = 78)	60.3	28 [14–42]	64.0	34 [30–42]	96.1	38 [20–52]	58.1	6 [3–18]	97.0	50 [42–56]	51.4	3 [2–16]	71.8	10 [5–21]
Differentiation	Well/Mod. (*n* = 29)	72.4^**a**^	27 [9–44]	69.0	37 [30–50]	86.2	45 [31–53]	44.4	9 [3–16]	84.6	53 [42–58]	40.7	3 [2–4]	73.1	9 [4–17]
	Poor (*n* = 78)	56.4	31 [19–42]	63.0	35 [30–42]	97.3	38 [22–55]	64.0	6 [3–19]	91.3	48 [40–54]	45.9	3 [2–14]	66.2	10 [5–21]
Vascular embolus	No (*n* = 60)	65.0	26 [14–45]	70.2	35 [31–43]	89.7^**d**^	45 [26–59]	55.0	10 [3–19]^**c**^	92.5	50 [42–57]	51.7	3 [2–13]	70.2	9 [5–20]
	Yes (*n* = 49)	59.2	31 [19–39]	55.3	37 [30–43]	100	39 [22–50]	65.9	3 [2–11]	86.0	48 [40–53]	40.0	5 [2–20]	66.7	11 [5–20]
pTNM stage	I-II (*n* = 40)	70.0	30 [14–47]	67.6	37 [33–45]	89.7^**d**^	46 [35–59]	59.0	10 [3–28]^**c**^	91.9	49 [41–56]	51.3	3 [2–8]	64.1	9 [5–19]
	III-IV (*n* = 59)	59.3	30 [19–40]	60.3	39 [32–43]	100	40 [22–53]	58.2	4 [2–17]	87.8	49 [41–54]	41.8	5 [2–20]	70.0	12 [5–28]
Depth of invasion	T_1-2_ (*n* = 25)	68.0	34 [11–52]	72.7	36 [31–44]	84.0^**e**^	48 [36–59]	73.9	10 [3–21]	86.4	52 [44–56]	47.8	2 [2–4]	70.8	9 [3–19]
	T_3_ (*n* = 53)	62.3	28 [17–41]	58.5	35 [30–43]	96.1	43 [28–53]	49.0	7 [2–24]	91.3	46 [36–56]	48.0	5 [2–18]	68.8	10 [5–18]
	T_4_ (*n* = 25)	60.0	30 [16–38]	62.5	42 [33–44]	100	31 [15–53]	66.7	5 [2–17]	90.9	49 [41–55]	36.0	8 [3–20]	66.7	14 [5–32]
Lymph node metastasis	N_0_ (*n* = 57)	68.4	26 [13–47]	67.3	35 [32–43]	89.1^**d**^	45 [22–58]	61.8	10 [3–20]	90.6	53 [43–57]^**c**^	46.4	2 [2–10]	67.3	9 [5–19]
	N_1-3_ (*n* = 51)	56.9	31 [21–41]	58.3	39 [30–44]	100	39 [26–52]	58.3	4 [2–16]	88.1	45 [37–52]	47.8	5 [2–20]	69.8	11 [5–23]
Distant metastasis	M_0_ (*n* = 77)	67.5^**a**^	30 [14–44]	64.4	37 [32–43]	92.0	42 [24–56]	60.0	8 [3–19]	91.5	49 [41–56]	46.7	3 [2–13]	68.1	9 [5–17]^**c**^
	M_1_ (*n* = 31)	51.6	30 [21–40]	60.0	36 [30–43]	100	43 [24–53]	60.7	6 [2–17]	83.3	49 [40–53]	48.1	6 [3–20]	69.2	15 [9–28]

* %; [25-75%], 25%-75% percentiles; ^a,^ Pearson’s Chi-square test, *miR-9-1* for age, differentiation, distant metastasis: *χ*^*2*^ = 7.924, 2.271, 2.402, and *P =* 0.005, 0.132, 0.121, respectively; ^b,^ Student *t*-test: *miR-9-3* for location [42 ± 2 (*Mean* ± *SD*) vs 34 ± 1], *t* = 3.303, *P =* 0.002; *miR-137* for age [34 ± 3 vs 44 ± 3], *t* = −2.651, *P =* 0.009; for location [46 ± 3 vs 37 ± 2]*, t* = 2.103, *P =*0.038; ^c,^ Mann–Whitney *U*-test: *miR-34b* for pTNM stage/ vascular embolus, *U* = 239.000/320.000, *P =* 0.028/0.025, *miR-200b* for lymph node metastasis, *U* = 627.000, *P =* 0.021; *miR-210* for distant metastasis, *U* = 301.000, *P =* 0.048; ^d,^ Fisher’s exact test: *miR-137* for vascular embolus/pTNM/lymph metastasis, *P =* 0.031/0.024/0.028; *miR-200b* for location, *P =* 0.002; ^e,^*Trend* test: *miR-137* for depth of invasion, *χ*^*2*^ = 6.330, *P =* 0.012.

**Figure 4:**
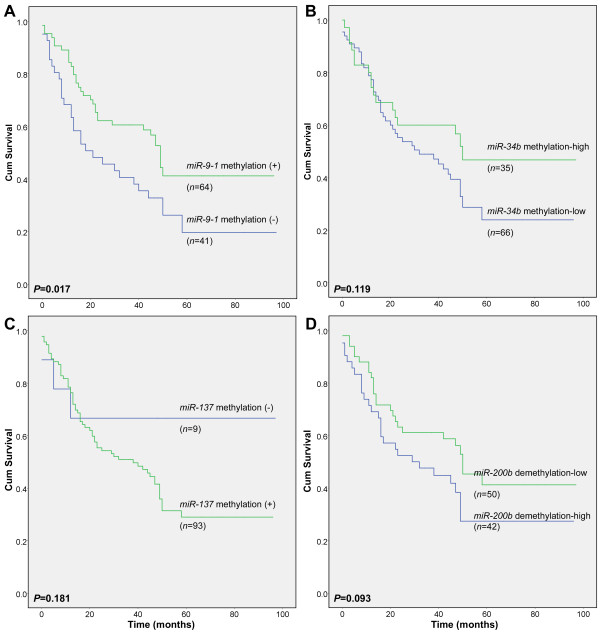
**Overall survival curves of GC patients with different methylation status of 4 miR CpG islands in Kaplan-Meier analysis.** (**A**) *miR-9-1* methylation used as the classifier; (**B**) *miR-34b* methylation used as the classifier; (**C**) *miR-137* methylation used as the classifier; (**D**) *miR-200b* demethylation used as the classifier.

Kaplan-Meier analysis showed that the overall survival of GC patients with *miR-9-1* methylation was significantly longer than in those without methylation (*P =* 0.017; Figure 4A). Similarly, the overall survival of GC patients with high *miR-34b* methylation (the proportion of methylated *miR-34b* > 4%) was likely longer than those with low *miR-34b* methylation (*P =* 0.119; Figure 4B). In contrast, the overall survival of GC patients with *miR-137* methylation was shorter than for those without methylation, but was not statistically significant (*P =* 0.181; Figure 4C). Interestingly, the overall survival of GC patients with high levels of demethylated *miR-200b* (proportion of methylated *miR-200b* <45%) was shorter than for low demethylation patients (*P =* 0.093; Figure 4D). In addition to *miR-9-1* methylation, vessel embolus, pTNM stages, invasion, node and distant metastasis are also significant survival predictors in univariate analysis (Additional file 1: Table S7). After adjustment for age, sex, differentiation, location, preoperative chemotherapy, pTNM stage, and vascular embolus, *miR-9-1* methylation was still an adequate prognostic indicator in multivariate analysis (adjusted *P =* 0.014; hazard ratios = 0.475 [95% CI, 0.262–0.860]).

## Discussion

The expression patterns exhibited by the *miR* genes reflect cell lineages and differentiation status of tumor tissue [34]. Increasing attention is currently being focused on miRNAs due to their contribution to maintaining the pluripotency of cancer stem/initiating cells and their application potential as molecular therapy targets and biomarkers. However, the regulatory mechanisms of *miR* expression in cells and alterations of miRNA levels in tumors are far from clear. In the present study, we characterized the methylation status of 9 representative *miR* CpG islands in human gastric tissues with various pathological changes and found that abnormal methylation of 5 *miR* CpG islands (*miR-9-1*, *miR-9-3*, *miR-34b/c*, *miR-137*, and *miR-210*) and demethylation of *miR-200b* CpG island correlated with the development of GC. Furthermore, methylation of the *miR-203* and *miR-375* CpG islands might be one kind of host adaptations to gastric carcinogenesis. Most importantly, we found that methylation of all these CpG islands was inversely correlated with the expression of these *miR* genes in a panel of cultured cell lines. To the best of our knowledge, this is the first comprehensive study to illustrate the inverse relationship between the methylation of *miR* CpG islands and their expression.

Transcription of protein-coding genes is mainly regulated by transcription factors and the accessibility of their binding sites in the promoter regions. It is well known that methylation of CpG islands around the TSS can block transcription factor binding by decreasing the accessibility of these sites, thus inactivate gene expression epigenetically. Bioinformatic analysis shows that 129 of 721 (18%) of the human *miR* genes in the miRBase (Release 14.0) are CpG island-related (Additional file 1: Table S1). Of the CpG related *miR* genes, 50 locate within CpG islands and 9 closely flank CpG islands with a <600-bp CpG-sporadic interval; an additional 70 *miR* genes are near CpG islands with a 0.6 ~ 10-kb CpG-sporadic interval. It has been suggested that methylation of some *miR* CpG islands might be inversely correlated to their expression [12-17]. However, a solid relationship between *miR* methylation and expression has not been thoroughly established as only weak supporting evidence has been provided in many of the previous studies, as we have summarized for 9 tested *miR* genes/clusters (extragenic *miR-9-3*, *miR-137*, *miR-200b/200a/429*, *miR-203*, *miR-375*; intragenic *miR-9-1*, *miR-34b/c*, *miR-193b/365-1*, and *miR-210*) in this present study (Additional file 1: Table S2) [19-27]. One frequently used evidence is that methylated *miR* genes could be reactivated by inhibitors of DNA methyltransferase (5-aza-cytosine or 5-aza-deoxycytosine) because these cytosine analogues, which cannot be methylated in DNA, inhibit maintenance methylation through direct replacement of methylation targets (cytosine residues). However, this method is not adequate as methyltransferases inhibition that may indirectly reactivate transcription of both CpG island-free genes and methylated genes through DNA damage and repair pathways [35].

To investigate systemically if methylation of *miR* CpG islands represses transcription of *miR* genes, various cell lines and tissue samples with different methylation status of representative *miR* CpG islands must be used. Thus, instead of randomly selecting candidates from the 129 *miR* CpG islands, we first screened a set of CpG islands of *miR* genes that may be abnormally methylated during gastric carcinogenesis. Initially the methylation status of 9 *miR* CpG islands in human gastric tissues with various pathological changes was analyzed with DHPLC. As is consistent with others’ reports [14,15,19,25,27,36-40], *miR-9-1*, *miR-9-3*, *miR-34b*, *miR-137*, and *miR-375* methylation was observed in gastric carcinogenesis in the present study. We also observed methylation changes in *miR-200b*, *miR-193b*, *miR-203*, and *miR-210* CpG islands in the development of GCs that has not been previously reported. Interestingly, we found that the proportion of demethylated *miR-200b* gradually increased significantly in gastric tissues along with the severity of these changes. This suggests that *miR-200b* demethylation (or hypomethylation) may be involved in gastric carcinogenesis. In addition, *miR-203* and *miR-375* methylation increased gradually in gastritis and SM samples, but decreased in GC samples. This implies that *miR-203* and *miR-375* methylation is not a GC-specific event, but rather a host adaptation to gastric carcinogenesis.

Expression of some intragenic *miR* genes is coordinately regulated by the transcriptional mechanism of their host genes. However, a certain proportion of intragenic *miR* genes has their own TSS and transcribe in a host gene-independent pattern [9,41-43]. Unfortunately, the TSS sites have not been characterized for most extragenic/intergenic *miR* genes. It is reported that expression levels of some *miR* genes (including intragenic *miR-152* and *miR-34a/b/c* and extragenic *miR-203*, *miR-124-1/124-2*, *miR-129-2*, and *miR-181c*) inversely correlate with methylation of their corresponding CpG islands [11,13,14,16,27,38,44]. In the present study, we found that methylation or demethylation of all 7 tested *miR* CpG islands (GC-related *miR-9-1*, *miR-34b*, *miR-9-3*, *miR-137*, *miR-210*, *miR-200b* and host-related *miR-375*) was consistently, inversely correlated to a statistically significant level with their corresponding miRNA levels in a number of human cell lines *in vitro*. Such an inverse relationships could also be observed for the *miR-9-1*, *miR-9-3*, *miR-137*, and *miR-200b* CpG islands in gastric tissue samples *in vivo*. Although expression of intragenic *miR-9-1* is host gene-independent [9], we cannot exclude the possibility that the expression of *miR-34b* and *miR-210* genes may also be coordinately controlled by regulatory mechanisms of their host genes. An unbiased correlation study using a customized, oligo microarray to detect methylation of all *miR* CpG islands and expression of intragenic *miR* genes and their host genes in various cell lineages at different differentiation stages may be useful to clarify which *miR* genes may be expressed in a host-gene dependent way. Taken together, these data strongly suggest that the methylation status of *miR* CpG islands could play a crucial role in the regulation of the expression of the related *miR* genes.

Generally, transcriptional inactivation of a *miR* gene by methylation in a few cells should not lead to a visible decrease in the miRNA level in a tissue sample because the majority of cells will still exhibit normal miRNA expression. This help explain why a significant inverse methylation-expression relationship was not observed for miRNA-34b, miRNA-210, or miRNA-375 as the average proportion of methylated *miR-34b*, *miR-210*, or *miR-375* in SM and GC samples was relatively low (2% ~ 15%). Hypermethylation of genes in some cell populations and the concomitant over-expression of these genes in other cell populations are often reported in the same tissue samples with chronic inflammation [18]. Therefore, both the prevalence of *miR* methylation and the total mature miRNA levels may be useful predictors for *miR* inactivation and expression in tissue samples, especially in the case of low proportion of methylated *miR*. Although other factors certainly affect the miRNA expression levels in cells, the inverse relationship consistently observed in this present study between *miR* methylation and mature miRNA expression level in a number of monoclonal cell lines suggests that the cellular heterozygosity may account for the inconsistent methylation-expression relationship of some *miR* in tissue samples.

The *miR-9-1*, *miR-9-2*, and *miR-9-3* genes all encode the same mature miRNA-9 that affects cell migration and proliferation in a tumor type-specific pattern through the *NF-κB1SnailE-cadherin*[45,46]. Abnormal *miR-9-1* and *miR-9-3* methylation is frequently reported in many cancers including GCs [15,19,40]. In the present study, we found that the positive rate of *miR-9-1* and *miR-9-3* methylation for all 112 GCs was significantly higher than that in 50 non-malignant tissues collected from 37 gastritis patients and 13 healthy controls. In all the tested samples, the prevalence of *miR-9-3* methylation is significantly higher than that of *miR-9-1*. It has been reported that *miR-9-3* methylation correlates with poor clinical outcomes for GC patients [40]. However, our study failed to make the same correlation. In contrast, a significant higher of the positive rate and proportion of methylated *miR-9-1*, but not methylated *miR-9-3*, was observed in GCs than SMs. This data indicates that *miR-9-1* methylation may be a late cancer-specific event, while *miR-9-3* methylation may be an early field effect during gastric carcinogenesis. Using *miR-9-1* methylation as a biomarker for GC detection, we found a sensitivity and specificity 62% (69 of 112) and 96% (46 of 48) could be achieved, respectively. Because the positive rate of *miR-9-1* methylation in GCs or SMs from stage I ~ II GC patients was similar to that of stage III ~ IV GC patients (GCs: 70% versus 59%; SMs: 40% versus 25%), it would be clinically beneficial to determine if *miR-9-1* methylation in gastric juice or peripheral blood plasma could be used as a biomarker for prediction of malignant transformation of precancerous lesions of the stomach and early diagnosis of GCs. Although *miR-9-1* methylation does not significantly correlate with differentiation, local invasion, metastasis, and pTNM stages in the screening cohort, it does significantly correlate with a longer overall survival of GC patients in univariate and multivariate analysis. Combination with the methylation status of other *miR* genes failed to strengthen the prediction power of *miR-9-1* methylation on the survival of these patients. Therefore, *miR-9-1* methylation might be an independent predictor of survival.

*miR-137* has been reported to target the CtBP1, a co-repressor of various tumor suppressor genes [47]. Many studies have reported *miR-137* methylation in head and neck and colorectal carcinomas [22,23,48,49]. We found that *miR-137* methylation was also very common among gastritis lesion, SM, and GC samples (63% ~ 96%), and the positive rate of *miR-137* methylation increased gradually in gastric tissues along with the severity of pathological changes. Therefore, *miR-137* methylation may be another early epigenetic event that occurs during gastric carcinogenesis. Because the proportion of methylated *miR-137* showed a positive correlation with the invasiveness of GCs, *miR-137* methylation may also affect the progression of the tumor. When compared to the GC patients without *miR-137* methylation, GC patients with *miR-137* methylation were more likely to have a poor overall survival; however, this effect was not statistically significant. This is consistent previous study that has reported that *miR-137* methylation (by MSP) correlated to the overall survival of 67 patients with head and neck carcinomas [49].

*miR-34b* and *miR-34c* constitute a *miR* gene cluster. Previous studies have suggested that mature miRNA-34b and miRNA-34c are targets of P53 [50]. As a tumor suppressor gene, *miR-34b/c* methylation has also frequently been reported in many carcinomas including GCs [14,51,52]. *miR-34b* methylation has been reported to relate to the invasiveness of non-small lung carcinoma, colorectal carcinoma, GC, and melanoma [53-55]. In the present study, we did not find a significant correlation between *miR-34b* methylation and GC invasion; however, a higher proportion of methylated *miR-34b* was observed in stage I ~ II GCs than in stage III ~ IV GCs (*P =* 0.025). The overall survival of GC patients with methylated *miR-34b* (the proportion > 4%) was likely to be longer than in those patients without the methylation. In addition, we found that the prevalence of *miR-34b* methylation between SM and GC samples was similar; this is consistent with a recent report that *miR-34b* methylation might be an early field-effect in the development of GCs [55].

The *miR-200b* gene is located in the *miR-200b/miR-200a*/*miR-429* cluster. Its function is related to the processes of epithelium-mesenchymal transition (EMT) by targeting ZEB1 and ZEB2 and results in *E-cadherin* silencing [56-58]. There is a CpG island upstream of this cluster which contains a 4-kb CpG-sporadic interval. It has been reported that *miR-200a/b* is hypomethylated and over-expressed in pancreatic cancers and that circulating miRNA-200 may be used as a cancer biomarker. The positive relationship between *miR-200b* demethylation and over-expression has also been previously found [59]. In the present study, we observed that full demethylation of the *miR-200b* CpG island was a common field event in both GC and SM samples. Because the proportion of demethylated *miR-200b* in gastritis/normal biopsies was significantly lower than that in SM and GC samples, *miR-200b* demethylation might be another potential GC biomarker.

*miR-210* is a hypoxia inducible gene which may inhibit cancer cell survival and proliferation through targeting FGFRL1 [60]. It has been reported that *miR-210* may directly bind to vacuole membrane protein 1 (VMP1) and promote cancer metastasis [61]. *miR-210* methylation has been previously reported to be presented in glioma [62]. We found that *miR-210* methylation not only correlated with the severity of gastric pathogenesis, but also correlated with *H*. *pylori* infection. According to the possible cause effect relationship between *H*. *pylori* infection and the development of GCs, *miR-210* inactivation by DNA methylation may play a role in gastric carcinogenesis.

*miR-375* promotes cancer cell proliferation through RASD1-ERα pathway [63]. methylation has been previously reported in melonoma [64], breast cancer [63], gastric carcinoma [39] and hepatocellular carcinoma [12]. However, our present study found that for the 106 patients studied, there was a significant higher positive rate and proportion of methylated *miR-375* present in SMs compared to GCs. Because significant correlation between *miR-375* methylation and *H. pylori* infection was not observed among the tested gastric samples, we suggest that *miR-375* methylation might be a unique host adaptation to the development of GCs.

Previous studies have shown that *miR-203* methylation drives *H. pylori*-associated gastric lymphomagenesis [65]. *miR-203* and *miR-193b* methylation has also been reported in hepatomas and prostate cancer, respectively [12,37]. We observed that the positive rate of *miR-203* and *miR-193b* methylation increased in normal, gastritis, and SM tissues, but decreased in GCs as did *miR-375* methylation pattern. However, correlation between *H. pylori* infection and *miR-203* and *miR-193b* methylation was not found in any of the gastric tissue samples that were studied. Therefore, we hypothesize that *miR-203* and *miR-193b* methylation may also be a host adaptation to the development of gastritis or GCs.

## Conclusions

In conclusion, alteration of methylation status of 6 of 9 tested *miR* CpG islands was characterized in gastric carcinogenesis. *miR-210* methylation correlated with *H. pylori* infection. *miR-9-1* and *miR-137* methylation may be a GC-specific event. Methylation of *miR* CpG islands may significantly down-regulate their transcription regularly.

## Competing interests

The authors declare that they have no competing interests.

## Authors' contributions

YD participated in the design, conducted experiments, and wrote the manuscript; ZL contributed to the DHPLC assay development and gastric sample management; LG collected the clinical information; JZ extracted genomic DNA samples from tissues and detect *H. pylori* 23 S rDNA; B-DZ collected the gastric biopsy samples; JJ collected gastric carcinoma samples; DD designed and coordinated the study, analyzed data, and wrote the manuscript. All authors read and approved the final manuscript.

## Pre-publication history

The pre-publication history for this paper can be accessed here:

http://www.biomedcentral.com/1471-2407/12/249/prepub

## Supplementary Material

Additional file 1 **Figure S1.** Illustrations of 9 tested *miR*-hosting CpG islands. Open frame, location of pre-miRNA transcript. Open arrow, pre-miRNA transcript direction; Blue-underline, locations of the forward and reversed primer-matching regions used to detect methylation of CpG island with DHPLC and bisulfite sequencing; Purple vertical bars, CpG sites. **Figure S2**. DHPLC chromatogram of methylated and unmethylated *miR-9-1* in various cell lines. UV-detector; the partial denaturing temperature, 55.4**°**C; U, peak for the unmethylated PCR products; M, peak for the methylated PCR products; B, peripheral blood DNA; MB, *M.sssI*-methylated blood DNA. **Figure S3**. DHPLC chromatogram of methylated and unmethylated *miR-9-3* in various cell lines. UV-detector; the partial denaturing temperature, 58.5**°**C; U, peak for the unmethylated PCR products; M, peak for the methylated PCR products; B, peripheral blood DNA; MB, *M.sssI*-methylated blood DNA. **Figure S4**. DHPLC chromatogram of methylated and unmethylated *miR-34b* in various cell lines. UV-detector; the partial denaturing temperature, 56.8**°**C; U, peak for the unmethylated PCR products; M (partial or full), peak for the partially or fully methylated PCR products; B, peripheral blood DNA; MB, *M.sssI*-methylated blood DNA. **Figure S5**. DHPLC chromatogram of methylated and unmethylated *miR-210* in various cell lines. Fluorescence-detector; the partial denaturing temperature, 58.7**°**C; U, peak for the unmethylated PCR products; M, peak for the methylated PCR products; B, peripheral blood DNA; MB, *M.sssI*-methylated blood DNA. **Figure S6**. DHPLC chromatogram of methylated and unmethylated *miR-137* in various cell lines. Fluorescence-detector; the partial denaturing temperature, 58.3**°**C; U, peak for the unmethylated PCR products; M, peak for the methylated PCR products; B, peripheral blood DNA; MB, *M.sssI*-methylated blood DNA. **Figure S7**. DHPLC chromatogram of methylated and unmethylated *miR-375* in various cell lines. UV-detector; the partial denaturing temperature, 55.7**°**C; U, peak for the unmethylated PCR products; M, peak for the methylated PCR products; B, peripheral blood DNA; MB, *M.sssI*-methylated blood DNA. **Figure S8**. DHPLC chromatogram of methylated and unmethylated *miR-200b* in various cell lines. UV-detector; the partial denaturing temperature, 55.6**°**C; U, peak for the unmethylated PCR products; M, peak for the methylated PCR products; B, peripheral blood DNA; MB, *M.sssI*-methylated blood DNA. **Figure S9**. DHPLC chromatogram of methylated and unmethylated *miR-193b* in various cell lines Fluorescence-detector; the partial denaturing temperature, 56.5**°**C; U, peak for the unmethylated PCR products; M, peak for the methylated PCR products; B, peripheral blood DNA; MB, *M.sssI*-methylated blood DNA. **Figure S10**. DHPLC chromatogram of methylated and unmethylated *miR-203* in various cell lines. UV-detector; the partial denaturing temperature, 57.7**°**C; U, peak for the unmethylated PCR products; M, peak for the methylated PCR products; B, peripheral blood DNA; MB, *M.sssI*-methylated blood DNA. **Figure S11**. Distribution of *miR* methylation in various gastric mucosa samples with and without H. pylori infection. Each line represents methylation status of one *miR* gene and each column represents one sample. The proportion of methylated allele is displayed step-wisely: negative (white square), positive and <30% (light gray square), or 30%–60% (deep gray square) or >60% (black square). Not informative sample is marked as “ND”. The case with or without *H. pylori*-specific *23 S rDNA* was labeled with letter “p” (light green) and “n” (blue square). **Figure S12**. Comparison of levels of 7 mature miRNAs by quantitative RT-PCR in 20 pairs of fresh gastric carcinoma (GC) and the corresponding surgical margin (SM) tissue samples. The relative miRNA levels of the paired GC and SM samples from the same patient were linked by a line. **Table S1**. Classification of 129 CpG island-related human *miR* genes from miRBase (Release 14.0). **Table S2**. Summary of reported data on the inverse methylation-expression relationship for 9 representative *miR* CpG islands analyzed in the present study. **Table S3**. Primer sequences, amplicons, and PCR/DHPLC annealing/ denaturing temperatures used in the detection of the methylation status of 9 miR CpG islands. **Table S4**. Methylation status of 7 *miR* CpG islands in the paired gastric carcinoma (GC) and corresponding surgical margin (SM) samples from a total of 112 GC patients. **Table S5**. miRNA polyA primer for quantitative RT-PCR assays. **Table S6**. Comparison of the methylation status in 7 *miR* genes from the surgical margin (SM) samples of 112 GC patients with various clinicopathological characteristics. **Table S7**. Univariate analysis of the relationship between overall survival of GC patients and *miR-9-1* methylation or other clinicopathological characteristics. Click here for file
