# Supplementary material for: Characterization of human gastric carcinoma-related methylation of 9 miR CpG islands and repression of their expressions in vitro and in vivo
Source: BMC Cancer. 2012 Jun 15;12:249. doi: 10.1186/1471-2407-12-249 (PMC3517451; doi:10.1186/1471-2407-12-249)
Supplement: Additional file 1 — Figure S1. Illustrations of 9 tested miR-hosting CpG islands. Open frame, location of pre-miRNA transcript. Open arrow, pre-miRNA transcript direction; Blue-underline, locations of the forward and reversed primer-matching regions used to detect methylation of CpG island with DHPLC and bisulfite sequencing; Purple vertical bars, CpG sites. Figure S2. DHPLC chromatogram of methylated and unmethylated miR-9-1 in various cell lines. UV-detector; the partial denaturing temperature, 55.4°C; U, peak for the unmethylated PCR products; M, peak for the methylated PCR products; B, peripheral blood DNA; MB, M.sssI-methylated blood DNA. Figure S3. DHPLC chromatogram of methylated and unmethylated miR-9-3 in various cell lines. UV-detector; the partial denaturing temperature, 58.5°C; U, peak for the unmethylated PCR products; M, peak for the methylated PCR products; B, peripheral blood DNA; MB, M.sssI-methylated blood DNA. Figure S4. DHPLC chromatogram of methylated and unmethylated miR-34b in various cell lines. UV-detector; the partial denaturing temperature, 56.8°C; U, peak for the unmethylated PCR products; M (partial or full), peak for the partially or fully methylated PCR products; B, peripheral blood DNA; MB, M.sssI-methylated blood DNA. Figure S5. DHPLC chromatogram of methylated and unmethylated miR-210 in various cell lines. Fluorescence-detector; the partial denaturing temperature, 58.7°C; U, peak for the unmethylated PCR products; M, peak for the methylated PCR products; B, peripheral blood DNA; MB, M.sssI-methylated blood DNA. Figure S6. DHPLC chromatogram of methylated and unmethylated miR-137 in various cell lines. Fluorescence-detector; the partial denaturing temperature, 58.3°C; U, peak for the unmethylated PCR products; M, peak for the methylated PCR products; B, peripheral blood DNA; MB, M.sssI-methylated blood DNA. Figure S7. DHPLC chromatogram of methylated and unmethylated miR-375 in various cell lines. UV-detector; the partial denaturing temperature, 55.7°C [file 1471-2407-12-249-S1.doc]

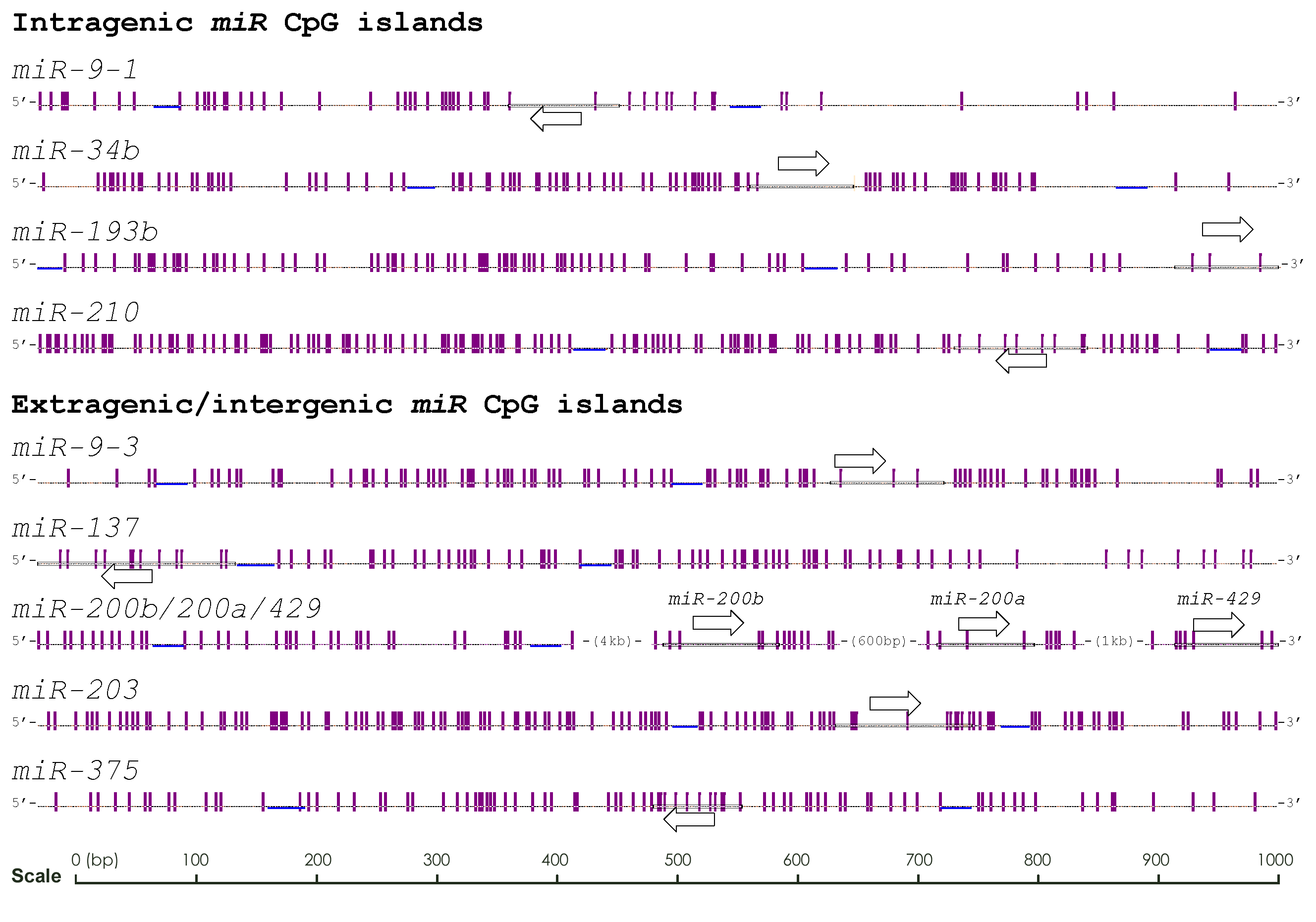


**Supplementary Fig. 1**. Illustrations of 9 tested *miR*-hosting CpG islands. Open frame, location of pre-miRNA transcript. Open arrow, pre-miRNA transcript direction; Blue-underline, locations of the forward and reversed primer-matching regions used to detect methylation of CpG island with DHPLC and bisulfite sequencing; Purple vertical bars, CpG sites; Scale, numerical base scale

**Supplementary Fig. 2**. DHPLC chromatogram of methylated and unmethylated *miR-9-1* in various cell lines. UV-detector; the partial denaturing temperature, 55.4°C; **U**, peak for the unmethylated PCR products; **M**, peak for the methylated PCR products; **B**, peripheral blood DNA; **MB**, *M.sssI*-methylated blood DNA

**U**

**M**

# U

**Supplementary Fig. 3**. DHPLC chromatogram of methylated and unmethylated *miR-9-3* in various cell lines. UV-detector; the partial denaturing temperature, 58.5°C; **U**, peak for the unmethylated PCR products; **M**, peak for the methylated PCR products; **B**, peripheral blood DNA; **MB**, *M.sssI*-methylated blood DNA

**U**

**M**

**Supplementary Fig. 4.** DHPLC chromatogram of methylated and unmethylated *miR-34b* in various cell lines. UV-detector; the partial denaturing temperature, 56.8°C; **U**, peak for the unmethylated PCR products; **M (partial or full)**, peak for the partially or fully methylated PCR products; **B**, peripheral blood DNA; **MB**, *M.sssI*-methylated blood DNA

**U**

**M (partial)**

**M (full)**

**Supplementary Fig. 5**. DHPLC chromatogram of methylated and unmethylated *miR-210* in various cell lines. Fluorescence-detector; the partial denaturing temperature, 58.7°C; **U**, peak for the unmethylated PCR products; **M**, peak for the methylated PCR products; **B**, peripheral blood DNA; **MB**, *M.sssI*-methylated blood DNA

**U**

**M**

**Supplementary Fig. 6**. DHPLC chromatogram of methylated and unmethylated *miR-137* in various cell lines. Fluorescence-detector; the partial denaturing temperature, 58.3°C; **U**, peak for the unmethylated PCR products; **M**, peak for the methylated PCR products; **B**, peripheral blood DNA; **MB**, *M.sssI*-methylated blood DNA

**U**

**M**

**Supplementary Fig. 7**. DHPLC chromatogram of methylated and unmethylated *miR-375* in various cell lines. UV-detector; the partial denaturing temperature, 55.7°C; **U**, peak for the unmethylated PCR products; **M**, peak for the methylated PCR products; **B**, peripheral blood DNA; **MB**, *M.sssI*-methylated blood DNA

**U**

**M**

**Supplementary Fig. 8**. DHPLC chromatogram of methylated and unmethylated *miR-200b* in various cell lines. UV-detector; the partial denaturing temperature, 55.6°C; **U**, peak for the unmethylated PCR products; **M**, peak for the methylated PCR products; **B**, peripheral blood DNA; **MB**, *M.sssI*-methylated blood DNA

**U**

**M**

**Supplementary Fig. 9**. DHPLC chromatogram of methylated and unmethylated *miR-193b* in various cell lines. Fluorescence-detector; the partial denaturing temperature, 56.5°C; **U**, peak for the unmethylated PCR products; **M**, peak for the methylated PCR products; **B**, peripheral blood DNA; **MB**, *M.sssI*-methylated blood DNA

**U**

### M

**Supplementary Fig. 10**. DHPLC chromatogram of methylated and unmethylated *miR-203* in various cell lines. UV-detector; the partial denaturing temperature, 57.7°C; **U**, peak for the unmethylated PCR products; **M**, peak for the methylated PCR products; **B**, peripheral blood DNA; **MB**, *M.sssI*-methylated blood DNA

**U**

### M

**
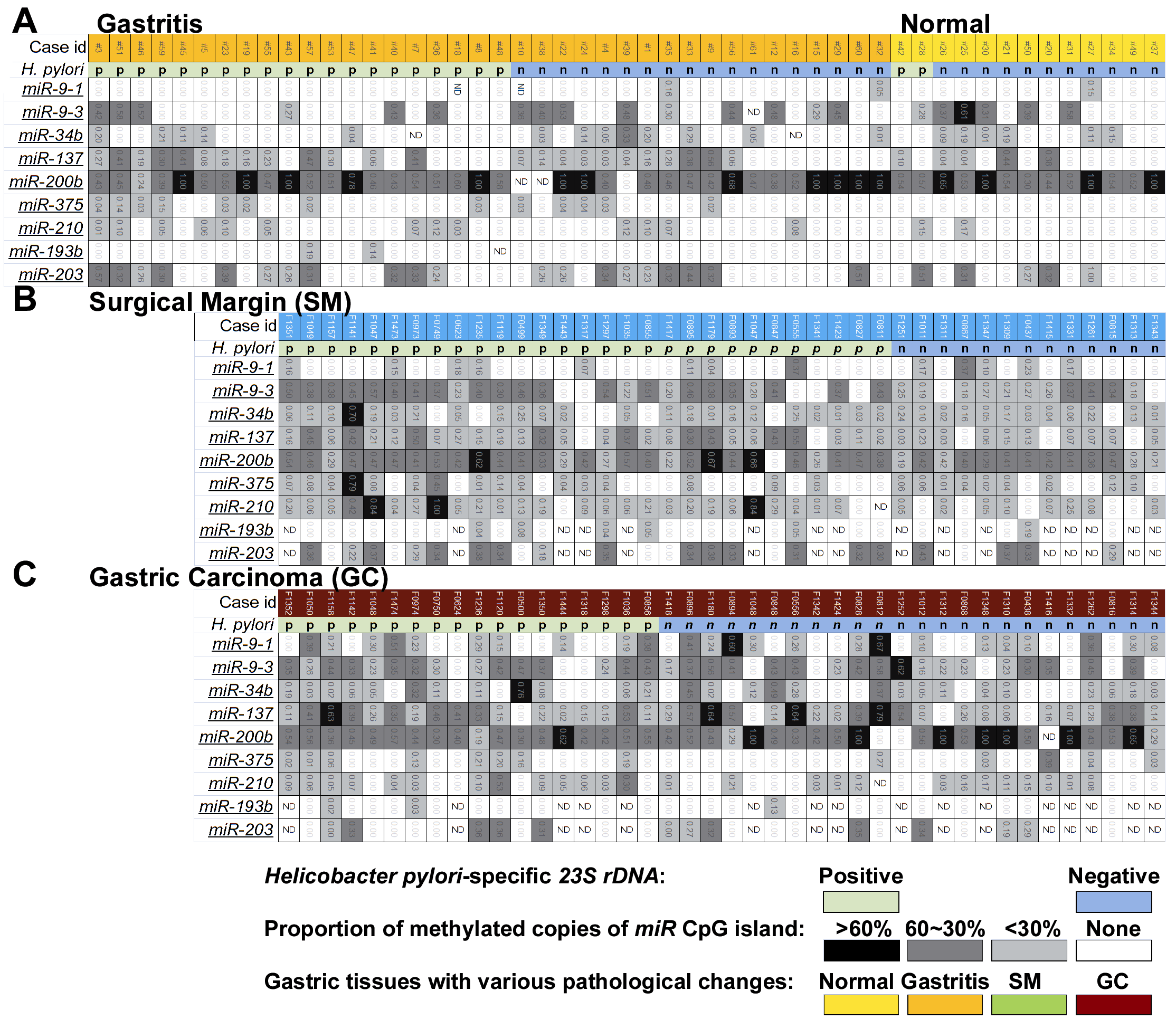
**

**Supplementary Fig. 11.** Distribution of *miR* methylation in various gastric mucosa samples with and without *H. pylori* infection. Each line represents methylation status of one *miR* gene and each column represents one sample. The proportion of methylated allele is displayed step-wisely: negative (⬜white), positive and <30% (⬛light gray), or 30%-60% (⬛deep gray) or >60% (⬛black). Not informative sample is marked as “ND”. The case with or without *H. pylori*-specific *23S rDNA* was labeled with letter “**p**” (⬛light green) and “**n**” (⬛blue).

**
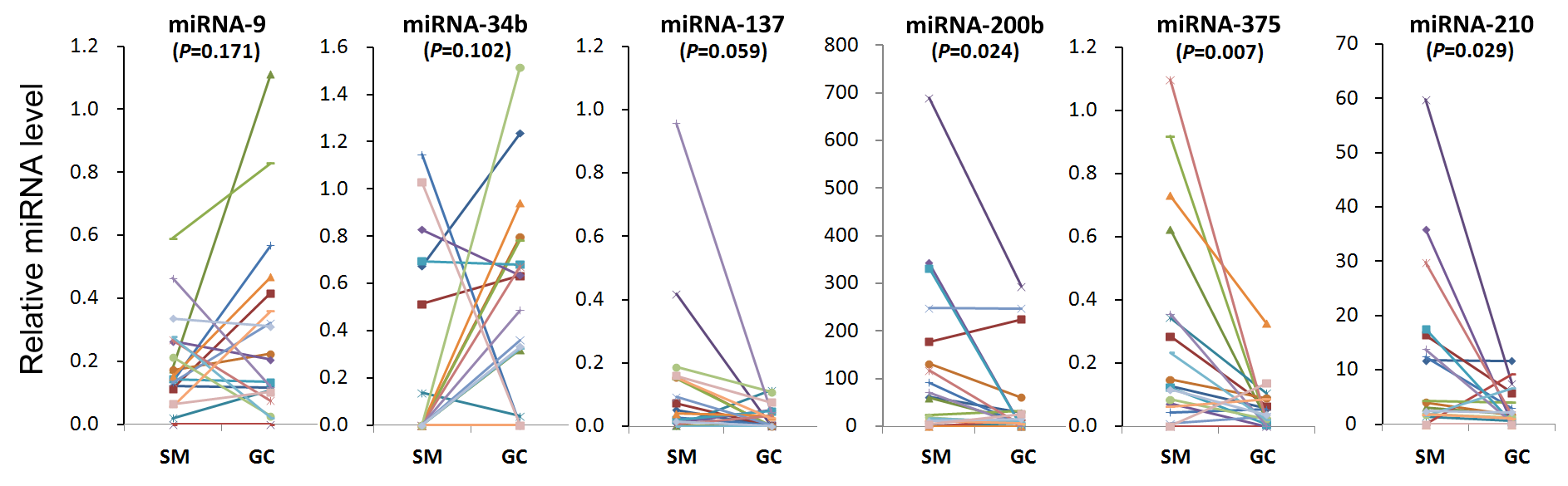
**

**Supplementary Fig. 12**. Comparison of levels of 7 mature miRNAs by quantitative RT-PCR in 20 pairs of fresh gastric carcinoma (GC) and the corresponding surgical margin (SM) tissue samples. The relative miRNA levels of the paired GC and SM samples from the same patient were linked by a line.

**Supplementary Table S1: Classification of 129 CpG island-related human *miR* genes from miRBase** (Release 14.0)

| 50 *miR* genes within CpG islands | | 9 *miR* genes flanking CpG islands (interval, <600-bp) | | 70 *miR* genes near CpG islands (interval, 0.6~10-kb) | |
| --- | --- | --- | --- | --- | --- |
| Single *miR* locus | *miR* gene cluster | Single *miR* locus | *miR* gene cluster | Single *miR* locus | *miR* gene cluster |
| *let-7i* | *miR-132*^a^ | *miR-153-1* | *miR-130b* | *miR-124-2* | *let-7a-1* |
| *miR-1237* | *miR-212* | *miR-345* | *miR-301b* | *miR-10a* | *let-7d* |
| *miR-124-1* | *miR-34b* | *miR-378* | *miR-191* | *miR-1-1* | *let-7f-1* |
| *miR-124-3* | *miR-34c* | *miR-596* | *miR-425* | *miR-196a-1* | *miR-15b* |
| *miR-129-2* | *miR-369* | *miR-9-1* |  | *miR-1227* | *miR-16-2* |
| *miR-137*^b^ | *miR-409* |  |  | *miR-1229* | *miR-195* |
| *miR-149* | *miR-410* |  |  | *miR-1236* | *miR-497* |
| *miR-152* | *miR-412* |  |  | *miR-1238* | *miR-17* |
| *miR-193a* | *miR-541* |  |  | *miR-146b* | *miR-18a* |
| *miR-196b* | *miR-656* |  |  | *miR-199b* | *miR-19a* |
| *miR-203* | *miR-658* |  |  | *miR-22* | *miR-19b1* |
| *miR-210* | *miR-659* (at upstream) |  |  | *miR-26b* | *miR-20a* |
| *miR-219-1* | *miR-939* |  |  | *miR-301a* | *miR-92a1* |
| *miR-219-2* | *miR-1234* (at upstream) |  |  | *miR-335* | *miR-18b* |
| *miR-339* | *miR-941-1* |  |  | *miR-483* | *miR-19b2* |
| *miR-375* | *miR-941-2* |  |  | *miR-505* | *miR-20b* |
| *miR-564* | *miR-941-3* |  |  | *miR-548b* | *miR-92a2* |
| *miR-572* |  |  |  | *miR-550-1* | *miR-106a* |
| *miR-574* |  |  |  | *miR-550-2* | *miR-363* |
| *miR-611* |  |  |  | *miR-554* | *miR-25* |
| *miR-615* |  |  |  | *miR-559* | *miR-93* |
| *miR-638* |  |  |  | *miR-561* | *miR-106b* |
| *miR-639* |  |  |  | *miR-578* | *miR-449a* |
| *miR-663* |  |  |  | *miR-580* | *miR-449b* |
| *miR-675* |  |  |  | *miR-589* | *miR-200b* |
| *miR-760* |  |  |  | *miR-607* | *miR-200a* |
| *miR-92b* |  |  |  | *miR-618* | *miR-429* |
| *miR-9-3* |  |  |  | *miR-627* | *miR-193b* |
| *miR-933* |  |  |  | *miR-637* | *miR-365-1* |
| *miR-935* |  |  |  | *miR-641* | *miR-181c* |
| *miR-943* |  |  |  | *miR-642* | *miR-181d* |
| *miR-126* |  |  |  | *miR-671* | *miR-182* |
| *miR-1225* |  |  |  | *miR-887* | *miR-183* |
|  |  |  |  | *miR-9-2* | *miR-96* |
|  |  |  |  | *miR-937* |  |
|  |  |  |  | *miR-940* |  |

^a^: *miR* gene listed in the neighboring blanks with the same color in the same column is a part of a *miR* gene cluster that locates within the same CpG island; ^b^: 9 underlined genes are the candidate genes tested in the present study.

**Supplementary Table 2: Summary of the methylation-expression relationship for 9 representative *miR*** CpG islands analyzed in the present study

| *miR* gene | Gene ID | Intragenic or Extragenic | Reported evidences on relationship between *miR* methylation and repression of its expression |
| --- | --- | --- | --- |
| *miR-9-3* | 407051 | Extragenic | No report |
| *miR-200b* | 406984 | Extragenic | No report |
| *miR-210* | 406992 | Hosted by *miR-210HG* | No report |
| *miR-9-1* | 407046 | Hosted by *C1for61* | 5-aza-cytosine-treated cell line, by COBRA/MSP and qRT-PCR ^19,20^ |
| *miR-193b* | 574455 | Hosted by *LOC100129781* | 5-aza-cytosine-treated cell line, by bisulfite sequencing and qRT-PCR ^21^ |
| *miR-203* | 406986 | Extragenic | 5-aza-deoxycytosine-treated cell line, by MSP and qRT-PCR ^22^ Cell lines, by COBRA and qRT-PCR ^12^ |
| *miR-137* | 406928 | Extragenic | Cell lines, by COBRA/MSP and qRT-PCR ^23,24^ **Gastric cancer tissues**, by MSP and qRT-PCR ^25^ |
| *miR-375* | 494324 | Extragenic | Cell lines, by COBRA and qRT-PCR ^12^ **Esophageal cancer tissues**, by MSP and qRT-PCR ^26^ |
| *miR-34b* | 407041 | Hosted by *LOC728196* | Cell lines, by COBRA/MSP and qRT-PCR ^23,21,27^ **Lung cancer tissues**, by MSP and qRT-PCR ^28^ |

**Supplementary Table 3: Primer sequences, amplicons, and PCR/DHPLC annealing/denaturing temperatures used in the detection of the methylation status of 9 miR CpG island**s

| *miR* gene | Primer sequence for *miR*-hosting  CpG islands | Size of PCR product (bp) | Number of CpG sites in the amplicon | PCR annealing temperature (°C) | DHPLC partial denaturing  temperature (°C) |
| --- | --- | --- | --- | --- | --- |
| *miR-137* | PF: 5’-taaggtagtatttttttgttttttttattt-3’ | 302 | 25 | 56.0 | 58.3 |
|  | PR: 5’-ccaaactactcaaaacctttcaatc-3’ |  |  |  |  |
| *miR-34b* | PF: 5’-aaggggaggtttggtatttttg-3’ | 596 | 59 | 56.4 | 56.8 |
|  | PR: 5’-cctccaaaaattttactttcctaac-3’ |  |  |  |  |
| *miR-375* | PF: 5’-attgaataggtagtataagagtatacggag-3’ | 567 | 58 | 54.3 | 55.7 |
|  | PR: 5’-taaaacctaacaacccaaaaactac-3’ |  |  |  |  |
| *miR-9-1* | PF: 5’-gaaattttttgggtttggatc-3’ | 493 | 36 | 56.4 | 55.4 |
|  | PR: 5’-ctcctcctcttatatcctctaatac-3’ |  |  |  |  |
| *miR-9-3* | PF: 5’-tagttttgaggtttttgagggtaga-3’ | 441 | 45 | 56.0 | 58.5 |
|  | PR: 5’-aaccaaaaaaaataaacaaacacac-3’ |  |  |  |  |
| *miR-203* | PF: 5’-gttgtagtagggtagggggt-3’ | 287 | 33 | 59.4 | 57.7 |
|  | PR: 5’-acccctaactataactctaactc-3’ |  |  |  |  |
| *miR-193b* | PF: 5’-gggtgtttaagtttttaggg-3’ | 646 | 69 | 50.0 | 56.5 |
|  | PR: 5’-cttctttttcctaacttaatattatc-3’ |  |  |  |  |
| *miR-200b* | PF: 5’-atgggagtttaggggatatatttgt-3’ | 330 | 23 | 56.4 | 55.6 |
|  | PR: 5’-tactctacctcaaccaaaatcaaac-3’ |  |  |  |  |
| *miR-210* | PF: 5’-tattggttgagggattaggttatttg-3’ | 538 | 57 | 56.0 | 58.7 |
|  | PR: 5’-tccctactaaccaaacaaacacacc-3’ |  |  |  |  |

**Supplementary Table 4: Methylation status of 7 *miR* CpG islands in the paired gastric carcinoma (GC) and corresponding surgical margin (SM) samples from a total of** 112 GC patients

| Subset of GC patients | *miR* CpG islands | Samples | *miR* methylation | | | | | | | |
| --- | --- | --- | --- | --- | --- | --- | --- | --- | --- | --- |
|  |  |  | Positive rate | | | Proportion (%) of methylated *miR* | | | | |
|  |  |  | Positive case no. /total case no. (%) | *χ^2^*-value | *P*-value | *Median* [25%-75%] (%) | *Mean* ± SD | *t*-value or *Z*-value | | *P*-value |
| Subset-1 | *miR-9-1* | SM | 9/28 (32.1) | 5.793 | 0.016 ^a^ | 17 [13-30] | 20 ± 4 | *t* = -3.039 | | 0.005 ^c^ |
|  |  | GC | 18/28 (64.3) |  |  | 29 [19-40] | 32 ± 5 |  | |  |
|  | *miR-9-3* | SM | 26/28 (92.9) |  | 1.000 ^b^ | 38 [26-44] | 36 ± 2 | *t* = 0.240 | | 0.812 ^c^ |
|  |  | GC | 26/28 (92.9) |  |  | 36 [26-43] | 35 ± 2 |  | |  |
|  | *miR-137* | SM | 25/26 (96.2) |  | 1.000 ^b^ | 15 [7-40] | 21 ± 3 | *t* = -2.652 | | 0.014 ^c^ |
|  |  | GC | 24/26 (92.3) |  |  | 36 [20-51] | 37 ± 4 |  | |  |
|  | *miR-34b* | SM | 22/28 (78.6) | 0.820 | 0.365 ^a^ | 11 [4-18] |  | *Z* = 0.000 | | 1.000 ^d^ |
|  |  | GC | 19/28 (67.9) |  |  | 10 [5-32] |  |  | |  |
|  | *miR-200b* | SM | 27/28 (96.4) |  | 1.000 ^b^ | 44 [40-47] | 44 ± 2 | *t* = -1.031 | | 0.312 ^c^ |
|  |  | GC | 27/28 (96.4) |  |  | 48 [40-53] | 49 ± 3 |  | |  |
|  | *miR-210* | SM | 22/27 (84.6) | 3.197 | 0.135 ^b^ | 11 [5-22] |  |  | | 0.043 ^d^ |
|  |  | GC | 16/27 (59.2) |  |  | 9 [4-16] |  |  | |  |
|  | *miR-375* | SM | 16/28 (57.1) | 4.667 | 0.031 ^a^ | 7 [4-14] |  |  | | 0.481 ^d^ |
|  |  | GC | 8/28 (28.6) |  |  | 17 [8-21] |  |  | |  |
| Subset-2 | *miR-9-1* | SM | 27/84 (32.1) | 13.785 | <0.001 ^a^ | 13 [10-18] |  | *Z* = -3.486 | | <0.001^d^ |
|  |  | GC | 51/84 (60.7) |  |  | 31 [14-44] |  |  | |  |
|  | *miR-9-3* | SM | 34/79 (43.0) | 2.052 | 0.152 ^a^ | 36 [26-46] |  | *Z* = -0.889 | | 0.374 ^d^ |
|  |  | GC | 43/79 (54.4) |  |  | 35 [32-43] |  |  | |  |
|  | *miR-137* | SM | 74/83 (89.2) |  | 0.247 ^a^ | 26 [13-42] | 26 ± 2 | *t* = -4.294 | | <0.001 ^c^ |
|  |  | GC | 79/83 (95.2) |  |  | 45 [25-54] | 38 ± 2 |  | |  |
|  | *miR-34b* | SM | 48/79 (60.8) | 0.648 | 0.421 ^a^ | 6 [2-13] |  | *Z* = -1.008 | | 0.313 ^d^ |
|  |  | GC | 43/79 (54.4) |  |  | 4 [2-18] |  |  | |  |
|  | *miR-200b* | SM | 67/71 (94.4) |  | 0.244 ^b^ | 48 [40-56] |  | *Z* = 0.000 | | 1.000 ^d^ |
|  |  | GC | 62/71 (87.3) |  |  | 49 [42-57] |  |  | |  |
|  | *miR-210* | SM | 58/76 (76.3) | 0.140 | 0.708 ^a^ | 6 [4-11] |  | *Z* = -0.992 | | 0321^d^ |
|  |  | GC | 58/76 (73.7) |  |  | 11 [5-23] |  |  | |  |
|  | *miR-375* | SM | 53/78 (67.9) | 4.500 | 0.034 ^a^ | 4 [2-14] |  | *Z* = -2.016 | | 0.044 ^d^ |
|  |  | GC | 40/78 (51.3) |  |  | 3 [2-8] |  |  | |  |
| (Total) | *miR-9-1* | SM | 36/112 (32.1) | 19.523 | 0.001 ^a^ | 15 [11-22] |  | *Z* = -4.137 | | <0.001^d^ |
|  |  | GC | 69/112 (61.6) |  |  | 30 [15-42] |  |  | |  |
|  | *miR-9-3* | SM | 60/107 (56.1) | 1.581 | 0.209 ^a^ | 37 [26-45] |  | *Z* = -0.738 | | 0.461 ^d^ |
|  |  | GC | 69/107 (64.5) |  |  | 35 [30-43] |  |  | |  |
|  | *miR-34b* | SM | 70/107 (65.4) | 1.079 | 0.261 ^a^ | 23 [11-41] | 11 ± 1 | *t* = -5.065 | | 0.001^c^ |
|  |  | GC | 62/107 (57.9) |  |  | 41 [23-54] | 14 ± 2 |  | |  |
|  | *miR-137* | SM | 99/109 (90.8) | 1.265 | 0.299 ^a^ | 7 [3-16] |  | *Z* = -0.949 | | 0.343 ^d^ |
|  |  | GC | 103/109 (94.5) |  |  | 7 [3-18] |  |  |  | |
|  | *miR-200b* | SM | 95/99 (94.9) | 1.803 | 0.179 ^a^ | 46 [40-55] |  | *Z* = -0.505 | 0.614 ^d^ | |
|  |  | GC | 89/99 (89.9) |  |  | 49 [41-56] |  |  |  | |
|  | *miR-210* | SM | 80/102 (78.4) | 2.065 | 0.151 ^a^ | 7 [4-15] |  | *Z* = -0.105 | 0.916 ^d^ | |
|  |  | GC | 71/102 (69.6) |  |  | 9 [5-19] |  |  |  | |
|  | *miR-375* | SM | 69/106 (65.1) | 8.411 | 0.004 ^b^ | 4 [3-13] |  | *Z* = -2.222 | 0.026 ^d^ | |
|  |  | GC | 48/106 (45.3) |  |  | 3 [2-14] |  |  |  | |

**^a.^**Pearson’s Chi-square test; ^b.^Fisher’s exact test; ^c^. Paired *t*-test; ^d^. Sign rank test

**Supplementary Table 5: miRNA polyA primers for quantitative RT-PCR assays**

| Primer | sequence |
| --- | --- |
| miRNA-200b forward primer | 5’-taata ctgcc tggta atgat gac-3’ |
| miRNA-210 forward primer | 5’-atatg tgcgt gtgac agcgg ctg-3’ |
| Universal reverse primer | 5’-gcgag cacag aatta atacg ac-3’ |
| RT adaptor primer | 5’-gcgag cacag aatta atacg actca ctata ggttt ttttt ttttv n-3’ |
| U6 forward primer | 5’-cgctt cggca gcaca tatac-3’ |
| U6 reverse primer | 5’-ttcac gaatt tgcgt gtcat-3’ |

**Supplementary Table 6: Comparison of the methylation status in 7 *miR* genes from the surgical margin (SM) samples of 112 GC patients with various** clinicopathological characteristics

| Clinicopathological features | | *miR-9-1* methylation | | *miR-9-3*  methylation | | *miR-137*  methylation | | *miR-34b* methylation | | *miR-200b* methylation | | *miR-375  methylation* | | *miR-210 methylation* | |
| --- | --- | --- | --- | --- | --- | --- | --- | --- | --- | --- | --- | --- | --- | --- | --- |
|  |  | Positive rate (%) | Proportion, *Median** [25-75%] | Positive rate (%) | Proportion, *Median* [25-75%] | Positive rate (%) | Proportion, *Median* [25-75%] | Positive rate  (%) | Proportion, *Median* [25-75%] | Positive rate (%) | Proportion, *Median* [25-75%] | Positive  rate (%) | Proportion, *Median* [25-75%] | Positive  rate (%) | Proportion, *Median* [25-75%] |
| Age | ≤60 (*n*=49) | 22.4 | 11 [8-16]^b^ | 56.2 | 36 [26-50] | 87.8 | 21 [10-41] | 66.7 | 8 [4-16] | 93.0 | 43 [39-50]^b^ | 69.6 | 5 [3-10] | 72.1 | 8 [5-27] |
|  | >60 (*n*=63) | 39.7 | 17 [11-29] | 55.9 | 38 [26-43] | 93.3 | 26 [12-42] | 64.4 | 6 [2-15] | 96.5 | 47 [41-58] | 61.7 | 4 [3-16] | 83.1 | 6 [4-10] |
| Sex | Male (*n*=80) | 36.2 | 14 [10-18] | 57.3 | 37 [27-44] | 89.6 | 23 [12-42] | 67.9 | 7 [4-16] | 98.6^c^ | 44 [38-56] | 59.5 | 5 [3-16] | 76.7 | 7 [5-16] |
|  | Female (*n*=32) | 21.9 | 23 [13-37] | 53.1 | 34 [23-46] | 93.8 | 22 [11-38] | 58.6 | 5 [3-16] | 86.7 | 46 [41-53] | 78.1 | 4 [2-11] | 82.8 | 6 [4-14] |
| Preoperative chemotherapy | No (*n*=100) | 31.0 | 14 [10-18] | 57.9 | 37 [27-44] | 90.7 | 24 [11-42] | 65.3 | 7 [3-15] | 94.4 | 44 [40-54] | 63.8 | 4 [3-14] | 78.3 | 7 [5-15] |
|  | Yes (*n*=9) | 33.3 | NA | 44.4 | 31 [19-43] | 88.9 | 21 [9-37] | 55.6 | 19 [4-24] | 100 | 54 [42-60] | 66.7 | 4 [1-9] | 71.4 | 4 [3-6] |
| Location | Cardic (*n*=34) | 24.9 | 11 [7-19] | 50.0 | 39 [28-45] | 90.9 | 29 [12-44] | 66.7 | 7 [3-20] | 93.8 | 50 [39-58] | 59.4 | 7 [2-22] | 87.1 | 8 [4-19] |
|  | Non-cardic (*n*=78) | 33.3 | 17 [11-24] | 58.7 | 37 [25-45] | 90.8 | 23 [10-40] | 64.9 | 7 [3-14] | 95.6 | 44 [40-53] | 67.6 | 4 [3-11] | 74.6 | 6 [4-14] |
| Differentiation | Well/Mod. (*n*=29) | 41.4 | 14 [9-22] | 51.7 | 27 [23-40]^b^ | 93.1 | 18 [10-39] | 74.1 | 6 [3-16] | 92.3 | 48 [41-58] | 63.0 | 6 [3-20] | 92.3 | 6 [4-10] |
|  | Poor (*n*=78) | 28.2 | 16 [11-23] | 60.3 | 38 [31-46] | 90.7 | 23 [13-42] | 64.0 | 8 [3-16] | 95.7 | 44 [40-54] | 63.5 | 4 [2-12] | 74.6 | 7 [4-18] |
| Vascular embolus | No (*n*=60) | 36.7 | 15 [10-24] | 45.6^a^ | 36 [26-45] | 89.7 | 27 [13-41] | 66.7 | 9 [3-16] | 98.1 | 46 [41-55] | 67.2 | 7 [4-18]^b^ | 71.9 | 6 [4-14] |
|  | Yes (*n*=49) | 26.5 | 16 [11-24] | 66.0 | 38 [26-46] | 91.7 | 23 [9-44] | 61.4 | 6 [3-18] | 90.9 | 44 [37-56] | 66.7 | 4 [2-7] | 85.7 | 8 [4-20] |
| pTNM stage | I-II (*n*=40) | 40.0 | 15 [10-22] | 43.2 | 40 [24-46] | 94.9 | 32 [14-44]^b^ | 66.7 | 6 [2-19] | 94.6 | 47 [41-56] | 59.0 | 6 [4-15] | 71.8 | 8 [4-18] |
|  | III-IV (*n*=59) | 25.4 | 18 [11-30] | 58.6 | 36 [25-44] | 87.9 | 22 [9-36] | 65.5 | 8 [3-17] | 94.0 | 45 [40-54] | 69.1 | 4 [2-15] | 78.0 | 7 [5-16] |
| Depth of invasion | T_1-2_  (*n*=25) | 30.0 | 17 [11-28] | 50.0 | 36 [28-43] | 100 | 29 [9-50] | 82.6 | 9 [3-18] | 95.5 | 50 [41-59] | 65.2 | 7 [3-12] | 83.3 | 8 [4-20] |
|  | T_3_ (*n*=53) | 32.1 | 14 [9-29] | 49.1 | 38 [25-45] | 86.3 | 23 [13-40] | 60.8 | 7 [2-18] | 95.7 | 47 [39-56] | 70.0 | 4 [3-19] | 68.8 | 7 [4-15] |
|  | T_4_ (*n*=25) | 36.0 | 17 [12-18] | 62.5 | 34 [23-47] | 88.0 | 25 [14-46] | 58.3 | 6 [4-15] | 90.9 | 44 [41-53] | 56.0 | 4 [2-10] | 85.7 | 7 [5-17] |
| Lymph node metastasis | N_0_ (*n*=57) | 42.1^a^ | 13 [9-18] | 49.1 | 36 [27-46] | 92.7 | 29 [12-43] | 60.0 | 6 [3-17] | 96.2 | 44 [41-55] | 67.9 | 5 [3-13] | 76.4 | 7 [4-15] |
|  | N_1-3_ (*n*=51) | 21.6 | 18 [13-30] | 60.4 | 38 [26-44] | 88.0 | 23 [12-39] | 70.8 | 8 [3-16] | 93.0 | 47 [38-56] | 65.2 | 4 [2-20] | 79.1 | 7 [5-17] |
| Distant metastasis | M_0_ (*n*=77) | 33.8 | 13 [10-19] | 49.3 | 36 [26-46] | 90.7 | 24 [12-41] | 64.0 | 7 [2-17] | 97.2 | 45 [40-55] | 64.0 | 5 [3-15] | 77.8 | 6 [4-13] |
|  | M_1_ (*n*=31) | 29.0 | 18 [12-33] | 66.7 | 39 [28-44] | 90.0 | 27 [13-53] | 67.9 | 5 [3-16] | 88.0 | 46 [42-56] | 74.1 | 4 [2-12] | 76.9 | 9 [5-27] |

* %; 25%-75% percentiles; ^a^, Pearson’s Chi-square test: *miR-9-1* for lymph node metastasis, *χ^2^* = 5.182, *P* = 0.023; *miR-9-3* for vascular embolus, *χ^2^* = 4.304, *P* = 0.038; ^b^. Mann- Whitney *U*-test: *miR-9-1*/*miR-200b* for age, *U* = 80.000/809.000, *P*=0.048/0.028; *miR-9-3* for differentiation, *U* = 211.000, *P* = 0.038; *miR-137* for pTNM, *U* = 697.000, *P* = 0.037; *miR-375* for vascular embolus, *U* = 398.000, *P*=0.024; ^c^, Fisher’s exact test: *miR-200b* for sex, *P*=0.027

**Supplementary Table 7: Univariate analysis of relationship between overall survival of GC patients and *miR-9-1*** methylation or other clinicopathological characteristics

|  |  | Case number | Survival months (*median)* | *P*-values |
| --- | --- | --- | --- | --- |
| *miR-9-1* methylation | Positive | 64 | 49 | 0.017 |
|  | Negative | 41 | 21 |  |
| Distant metastasis | Positive | 46 | 12 | 0.000 |
|  | Negative | 56 | 58 |  |
| Lymph node metastasis | Positive | 30 | 15 | 0.000 |
|  | Negative | 71 | not available |  |
| Depth of invasion | T_4_ | 71 | 30 | 0.000 |
|  | T_3_ | 48 | 23 |  |
|  | T_1-2_ | 53 | not available |  |
| Vascular embolus | Positive | 55 | 16 | 0.000 |
|  | Negative | 38 | 50 |  |
| pTNM stage | I-II | 23 | not available | 0.006 |
|  | III-IV | 51 | 17 |  |
| Age | ≤60 | 46 | 49 | 0.810 |
|  | >60 | 59 | 38 |  |
| Sex | Male | 73 | 40 | 0.533 |
|  | Female | 32 | 47 |  |
| Differentiation | Poor | 75 | 23 | 0.130 |
|  | Well/Mod. | 25 | 50 |  |
| Preoperative chemotherapy | No | 94 | 38 | 0.709 |
|  | Yes | 8 | 50 |  |
| Location | Cardiac | 30 | 40 | 0.604 |
|  | Non-cardiac | 75 | 44 |  |
